# Supplementary material for: Tunable unconventional spin orbit torque magnetization dynamics in van der Waals heterostructures
Source: Nat Commun. 2025 Sep 30;16:8722. doi: 10.1038/s41467-025-64109-3 (PMC12484963; doi:10.1038/s41467-025-64109-3)
Supplement: Supplementary file 1 — Supplementary Information [file 41467_2025_64109_MOESM1_ESM.pdf]

Supplementary Information

Tunable unconventional spin orbit torque magnetization dynamics in van der Waals heterostructures

Lalit Pandey[1,2*], Bing Zhao[1], Karma Tenzin[3,4], Roselle Ngaloy[1], Veronika Lamparská[3], Himanshu Bangar[1], Aya Ali[5], Mahmoud Abdel-Hafiez[6,7], Gaojie Zhang[8], Hao Wu[8], Haixin Chang[8], Lars Sjöström[1], Prasanna Rout[1], Jagoda Sławińska[3], Saroj P. Dash[1,2,9*]

[1]Department of Microtechnology and Nanoscience, Chalmers University of Technology, SE-41296, Göteborg, Sweden.
[2]Wallenberg Initiative Materials Science for Sustainability, Department of Microtechnology and Nanoscience, Chalmers University of Technology, SE-41296, Göteborg, Sweden.
[3]Zernike Institute for Advanced Materials, University of Groningen, Nijenborgh 3, 9747 AG Groningen, The Netherlands.
[4]Department of Physical Science, Sherubtse College, Royal University of Bhutan, 42007 Kanglung, Trashigang, Bhutan
[5]Center for Advanced Materials Research, Research Institute of Sciences and Engineering, University of Sharjah, Sharjah 27272, United Arab Emirates
[6]Department of Applied Physics and Astronomy, University of Sharjah, Sharjah, United Arab Emirates.
[7]Department of Physics and Astronomy, Uppsala University, Box 516, SE-751 20 Uppsala, Sweden.
[8]School of Materials Science and Engineering, Huazhong University of Science and Technology, 430074, Hubei, China.
[9]Graphene Center, Chalmers University of Technology, SE-41296, Göteborg, Sweden.

<u>**Content**</u>

Supplementary Note 1: Optical microscope images and atomic force microscopic measurements of $TaIrTe_4/Fe_3GaTe_2$ devices.

Supplementary Note 2: Angular dependent conductivity anisotropy measurements on TaIrTe4

Supplementary Note 3: Spin-orbit torque magnetization switching in $TaIrTe_4/Fe_3GaTe_2$ heterostructure with the current along the a and b axes of $TaIrTe_4$.

Supplementary Note 4: Anomalous Hall loop shift measurements with bias current in $TaIrTe_4/Fe_3GaTe_2$ devices.

Supplementary Note 5: Current induced change in coercivity and Joule heating effect information.

Supplementary Note 6: Planar Hall signal of Fe_3GaTe_2 and magnetic field dependent second harmonics behavior of $TaIrTe_4$.

Supplementary Note 7: Field-like torque components and anomalous Nerst effect in $TaIrTe_4/Fe_3GaTe_2$.

Supplementary Note 8: Field dependence and angle dependence second harmonics measurement on $TaIrTe_4/Fe_3GaTe_2$

Supplementary Note 9: Space group of bulk $TaIrTe_4$.

Supplementary Note 10: Calculated Fermi surfaces of bulk $TaIrTe_4$.

Supplementary Note 11: Spin texture and Rashba-Edelstein effect in bulk $TaIrTe_4$.

Supplementary Note 12: Comparison of spin-orbit torque parameters with state-of-the-art results

Supplementary Note 1: Optical microscope images and atomic force microscopic measurements of $TaIrTe_4/Fe_3GaTe_2$ devices

The $TaIrTe_4$ and Fe_3GaTe_2 flakes were exfoliated on top of each other inside the glovebox with an inert atmosphere to ensure a clean interface. The optical image showing the overlap region of $TaIrTe_4$ and Fe_3GaTe_2 flakes is provided in Fig. S1a, b, with the flakes outlined by dotted lines for clarity. The flakes almost have rectangular geometry. To prevent degradation over time, the stacked flakes are protected with a layer of Al_2O_3. The Hall bar geometry design is illustrated in Fig. S1c, d. The $TaIrTe_4/Fe_3GaTe_2$ devices were patterned into Hall-bar geometry using electron-beam lithography with Ti/Au contacts fabricated via electron-beam lithography and e-beam evaporation, as shown in Fig. S1e, f. The dimension of Hall bar structure for Dev1 is 4.2 μm×2.5 μm, Dev2 is 7.5 μm×6.6 μm, Dev3 is 4.4 μm×3.6 μm, for Dev4 is 3.9 μm × 1.6 μm and for Dev5 is 3.4μm×1.1 μm.

The thicknesses of the $TaIrTe_4$ and Fe_3GaTe_2 flakes are measured using atomic force microscopy (AFM). The height profiles from the AFM topography for Dev2 and Dev3 are presented in Fig. S1g, h. The thickness of the Fe_3GaTe_2 flake in Dev 2 and 3 is 49.3 nm and 46.8 nm, respectively, while the thickness of the $TaIrTe_4$ flake in Dev2 and Dev3 is 78.2 nm and 52.3 nm, respectively.

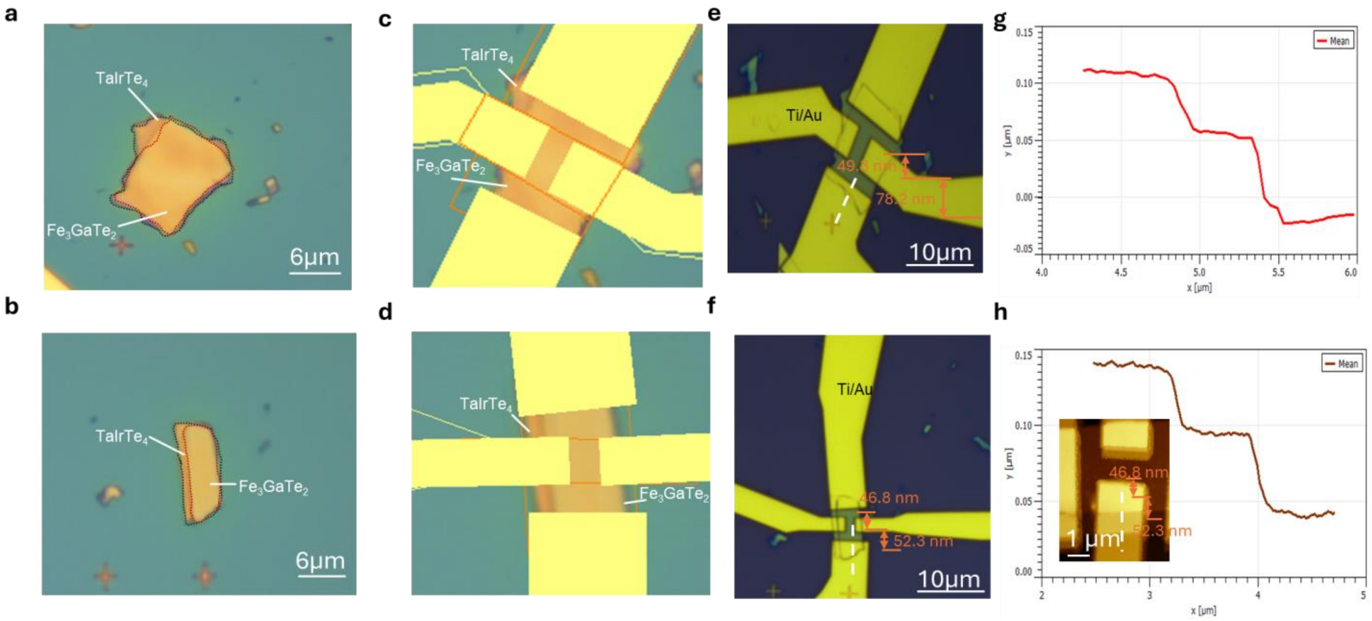

Supplementary Figure S1: Optical image and atomic force microscopic results of $TaIrTe_4/Fe_3GaTe_2$ Dev2 and Dev3. ***a,b,*** *Optical image of exfoliated $TaIrTe_4/Fe_3GaTe_2$ flakes with scale of 6 μm, depicting the areas where Devices 1 and 2 are fabricated. The flakes of $TaIrTe_4$ and Fe_3GaTe_2 are respectively outlined for clarity.* ***c,d,*** *Zoomed-in images of Autocad designs of Dev2 and Dev3, used in e-beam lithography.* ***e,f,*** *Optical image of $TaIrTe_4/Fe_3GaTe_2$ vdW heterostructure Hall bar Devices 2 and 3, with a scale bar of 10 μm.* ***g,h,*** *Atomic force microscopy thickness profiles of Fe_3GaTe_2 and $TaIrTe_4$ flakes used in Dev2 and Dev3. The thickness of Fe_3GaTe_2 flake is 49.3 nm and 46.8 nm for Dev2 and Dev3, respectively, whereas thickness of $TaIrTe_4$ flake is 78.2 nm and 52.3 nm for Dev2 and Dev3, respectively.*

Supplementary Note 2: Angular dependent conductivity anisotropy measurements on $TaIrTe_4$

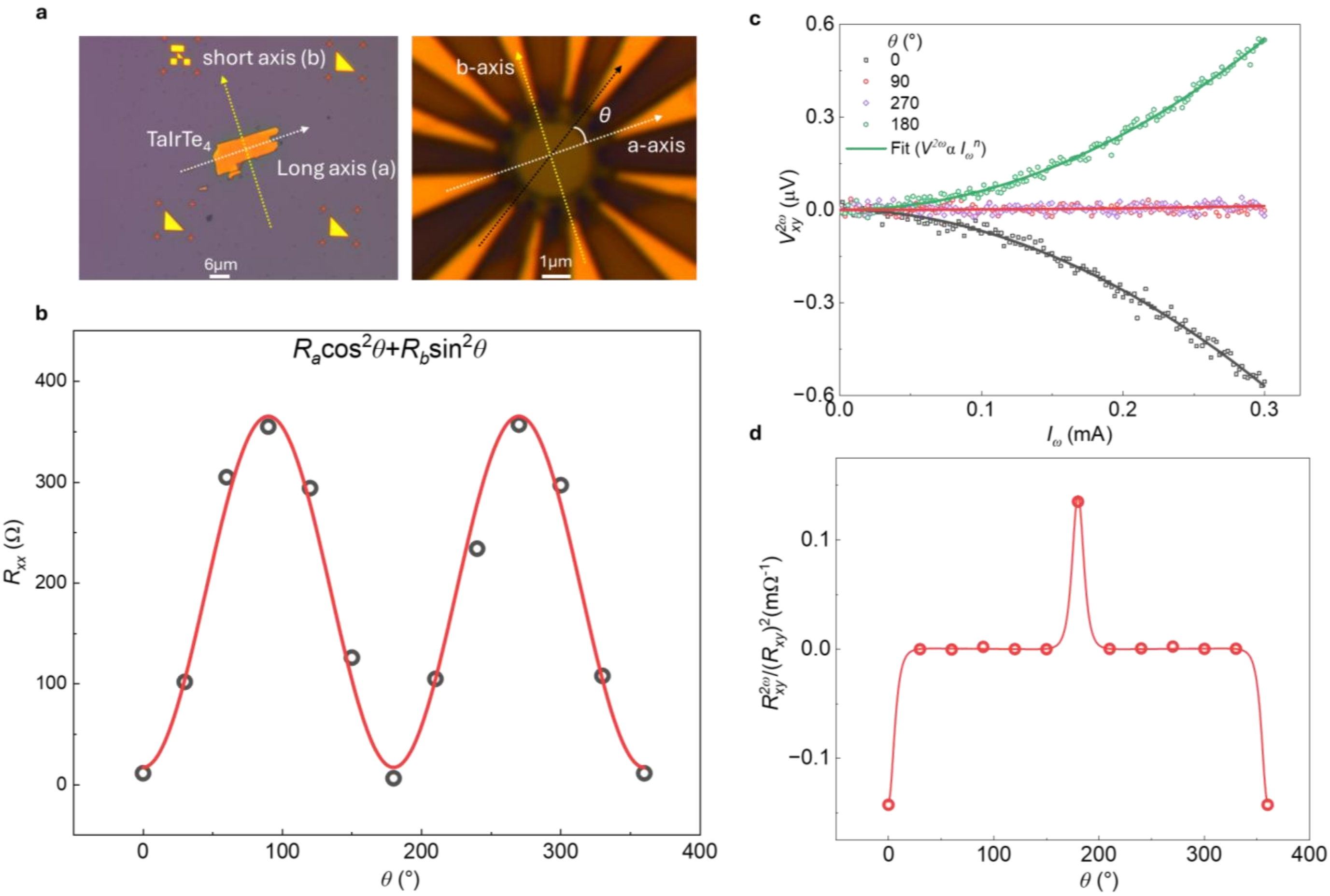

***Supplementary Figure S2: Angular dependence resistance measurements on TaIrTe4. a,** Optical image of $TaIrTe_4$ flake and circular disc device of TaIrTe4 depicting a-axis as long axis, whereas b-axis as short axis. **b,** Linear resistance (R_{xx}) as a function of θ. The curve is fitted with $R_{xx} = R_a \cos^2\theta + R_b \sin^2\theta$. **c,** $V_{xy}^{2\omega}$ as a function of I_ω measured at θ equal to 0° (a-axis), 90° (b-axis), 180° and 270°. The curve is fitted with power law equation ($V_{xy}^{2\omega}$ α I_ω^n), yielding n = ~2. **d,** $R_{xy}^{2\omega}/(R_{xy})^2$) as a function of θ. All voltages are measured via four-probe method.*

The crystallographic axis of $TaIrTe_4$ can be determined using polarized Raman spectroscopy (see our previous work ref. [1]) from which it can be concluded that the long axis of the exfoliated flakes reliably corresponds to the a-axis, while the short axis corresponds to the b-axis. Along with polarized Raman measurements, there are electrical means as well using which one can confirm the crystallographic axis[2]. The angular dependence of linear DC resistance R_{xx} in the circular disc device (see Fig. S2a) of $TaIrTe_4$ is shown in Fig. S2b. The R_{xx} vs θ fitted with using $R_{xx} = R_a \cos^2\theta + R_b \sin^2\theta$ where R_a and R_b are the resistance along the a-axis and b-axis, respectively and resistance anisotropy (r) defines R_a/R_b which reported to be smaller than 1 for $TaIrTe_4$. From the fitting, *r* found to be 0.047 ± 0.024 much smaller than 1, indicating a large conductivity anisotropy present in our $TaIrTe_4$ crystal, similar to previous reports[3,4]. The second harmonic transverse voltage $V_{xy}^{2\omega}$ as a function of I_ω injected along a-axis (θ ~ 0°, 180°) and b-axis (θ ~ 90°, 270°) is shown in Fig.S2c. The normalized nonlinear second harmonic transverse resistance as a function of θ is presented in Fig. S2d. The angular dependence fitted with $R_{xy}^{2\omega}/(R_{xy})^2 = A\cos\theta \frac{d_{12}r^2\cos^2\theta+(d_{11}-2d_{26}r^2)Sin^2\theta}{(\sin^2\theta+rcos^2\theta)^2}$,where d_{ij} are second order susceptibility tensor and *A* is normalized nonlinear amplitude. All three experimental results demonstrated in Fig. S2c-d further strengthen that conclusion that long axis of $TaIrTe_4$ correspond to a-axis whereas *b-axis as short axis.*

Supplementary Note 3: Spin-orbit torque magnetization switching in TaIrTe$_4$/Fe$_3$GaTe$_2$ heterostructure with the current along the a and b axes of TaIrTe$_4$

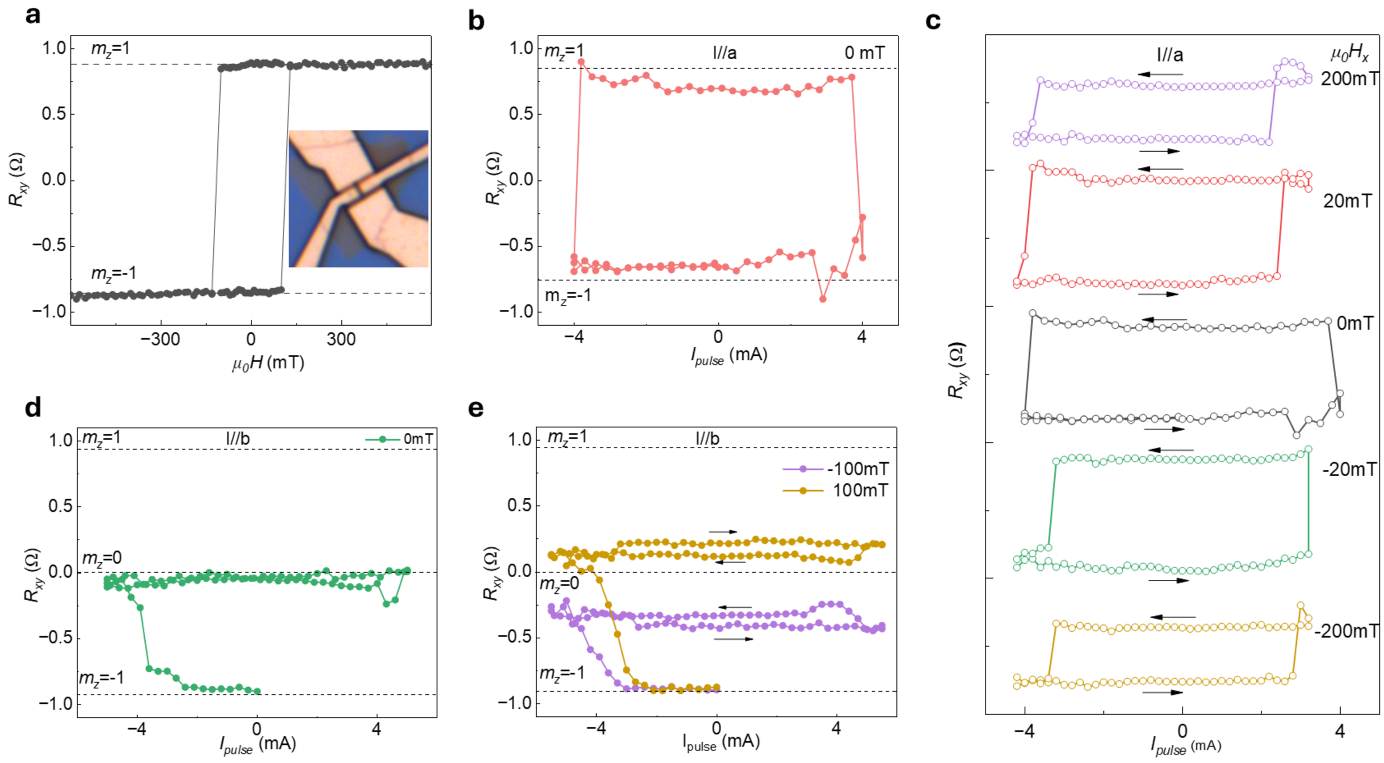

Supplementary Figure S3: Spin-orbit torque magnetization switching in TaIrTe$_4$/Fe$_3$GaTe$_2$ heterostructure at room temperature (Dev4). a. *Anomalous Hall effect (R_{xy} vs $\mu_0 H$) of the TaIrTe$_4$/Fe$_3$GaTe$_2$ heterostructure in Dev4, showing the magnetic field sweep at 300 K. Magnetization states corresponding to R_{xy} are indicated by dotted lines for $m_z = \pm 1$. The inset shows the optical image of Hall bar device.* ***b.*** *Field-free, fully deterministic switching is achieved with a ±4 mA pulse current, with a 200 μA read current used to measure the magnetization states at 300 K. The current is applied along the a-axis of TaIrTe$_4$ (I_{pulse} ∥a), keeping the external magnetic field set to zero.* ***c.*** *Current-driven magnetization switching of TaIrTe$_4$/Fe$_3$GaTe$_2$ under different bias fields parallel to the sample surface and current (H_x).* ***d.*** *Non-deterministic switching is observed when the current is applied along the b-axis of TaIrTe$_4$ (I_{pulse} ∥b), resulting in switching that does not exhibit deterministic behavior at zero magnetic field.* ***e.*** *Current-driven magnetization switching (I_{pulse} ∥b) probed by Hall resistance at 300 K under an in-plane magnetic field of ±100 mT. A small switching loop with a percentage of approximately 5% is observed.*

Figure S3a displays the anomalous Hall loop for Dev4, where R_{xy} is ~ 0.9 Ω and the coercivity is approximately 150 mT. The magnetization states are represented as $m_z = \pm 1$, corresponding to the anomalous Hall resistance, are indicated by a dotted line in the figure. The magnetization switching experiments were conducted at room temperature with current *I* applied along the a- and b- crystallographic axes of TaIrTe$_4$.

When a pulse current of ±4 mA is applied along the a-axis of TaIrTe$_4$, the magnetization states switch from $m_z = +1$ to $m_z = -1$ and vice versa (Fig. S3b). In contrast, when a pulse current of -4 mA is applied along the b-axis of TaIrTe$_4$, the magnetization only switches from $m_z = +1$ to $m_z = 0$ and remain in $m_z = +0$ even if the pulse current exceeds ±4 mA (Fig. S3d). This type of switching, where the magnetization does not fully switch, is classified as non-deterministic[5,6].

Additionally, deterministic spin-orbit torque (SOT) switching can be affected by an external in-plane magnetic field parallel to the current direction. The external in-plane magnetic field (H_x) can disrupt the symmetry of deterministic SOT switching. We observed that a field even up to H_x = 200 mT has minimal effect on the SOT switching signal. Notably, the switching polarity remains unchanged up to 200 mT, demonstrating the robustness of the out-of-plane spin polarization in TaIrTe$_4$ against the external magnetic field (Fig. S3c). In conventional SOT systems, where magnetization switching is driven solely by in-plane spin currents, the switching polarity typically reverses abruptly with H_x (Fig. S3e)[6]. However, this was not observed in our

experiments, suggesting that the contribution of τ_{DL}^{z} from $TaIrTe_4$ is significantly larger than τ_{DL}^{XY} in the magnetization dynamics of Fe_3GaTe_2.
Furthermore, in this device, we estimated the SOT efficiency (ε_{SOT}) to be 1.24, with the switching current density ($J_{switch} = I_{TIT}^{eff}$/t×w) calculated as $4.15 \times 10^{10} \mathrm{Am}^{-2}$, here I_{TIT}^{eff} is effective current pass through $TaIrTe_4$ as calculated using parallel resistor model[7,8], t is thickness of $TaIrTe_4$ and w is width of the Hall bar. Similarly, from the device parameters, we calculated the out of plane spin Hall conductivity (σ^{Z}_{SH}) in this device to be 1.16×10^{6} ħ/2e (Ωm)$^{-1}$ and switching power density P to be $1.832 \times 10^{15}\ \frac{\mathrm{W}}{\mathrm{m}^3}$. Moreover, when current is applied along b-axis of $TaIrTe_4$, only ~5% switching is observed even at H_x ~ 100mT, which give SOT efficiency due to the current induced in-plane spin polarized in $TaIrTe_4$ (b-axis) is 0.062 and an in-plane spin Hall conductivity σ^{XY}_{SH} ~ 5.82×10^{4} ħ/2e (Ωm)$^{-1}$.

In our study, we observed complete field-free switching of a 50 nm thick Fe_3GaTe_2 flake, an order of magnitude thicker than the Fe_3GaTe_2 used in literature[5,9–11]. The magnetization switching of 50 nm Fe_3GaTe_2 results in an order of magnitude larger SOT efficiency estimation. This finding is particularly intriguing, as spin current transfer from spin-orbit materials (SOMs) to adjacent ferromagnets is typically constrained by the spin diffusion length (λ_s). The successful switching in thicker flakes suggests the possible contribution of self-induced spin-orbit effects in Fe_3GaTe_2[12,13], which is also recently reported in similar 2D ferromagnetic systems such as Fe_3GeTe_2[14–20] and $Fe_{2.5}Co_{2.5}GeTe_2$[21]. Moreover, out of plane spin Hall conductivity in $TaIrTe_4/Fe_3GaTe_2$ is much larger than its in-plane counterparts, which is contrary to the result observe in $TaIrTe_4/Ni_{80}Fe_{20}$[1] in which σ^{Z}_{SH} is almost of same order as σ^{XY}_{SH} in line with the theoretical calculations. This different behavior of $TaIrTe_4/Fe_3GaTe_2$ suggests possible contribution of spin Hall effect in Fe_3GaTe_2 itself[12,13] along with interfacial and orbitals effects which may introduce extra states that leads to results deviating from theoretical calculations.

Furthermore, during the magnetization reversal in the pulsed current-induced magnetization switching measurements, there is some fluctuation in the R_{xy} value (Figs. 5c,d and Figs. S3b,c). Such fluctuations can originate from various factors including[22–25] (i) Joule heating effect and thermal fluctuations: The applied pulsed current can increase the temperature of the device due to Joule heating which can reduced the free energy barrier between the two-magnetization state, causing small deviations in R_{xy}. This effect will be more profound when the free energy barrier between two magnetic states becomes comparable to thermal energy of the device. We have estimated the device's temperature near the switching current, which is around 350 K (see section S5), close to the Curie temperature of Fe_3GaTe_2 (i.e., ~370 K). (ii) Multidomain nucleation: Application of large pulse current can enhance the possibility of local variation of magnetic properties such as magnetic coercivity, anisotropy and remanence value across the sample. Such effect can induce multiple domain nucleation and domain wall propagation leading to non-smooth magnetization reversal process, thereby arises fluctuations in R_{xy}. (iii) Improper Hall bar geometry: Due to slightest misalignment of Hall bar voltage electrode, the longitudinal resistance (R_{xx}) signal may superimpose over transverse resistance (R_{xy}). The R_{xx} signal tends to increase with pulse current due to Joule heating and R_{xx} should also abruptly change during magnetization reversal due to sudden spike of magnetoresistance. (iv) Spin-orbit torque dynamics: The magnetization dynamics of ferromagnet during switching can involve transient, non-uniform precession of the magnetic moment vector, leading to transient magnetic states, hence contributing to observed fluctuations. (v) Other measurement artifacts and stochastic process: Electrical noise in the measurement setup, such as Johnson-Nyquist noise, electromagnetic interferences, etc, can contribute to recorded fluctuations in R_{xy}. Magnetization reversal is inherently stochastic process and near critical current it can be influenced with even the presence of small external fields and manifests as fluctuation in R_{xy} value.

Supplementary Note 4: Anomalous Hall loop shift measurements with bias current in $TaIrTe_4/Fe_3GaTe_2$ devices

Anomalous Hall effect (AHE) loop shift measurements were conducted on Devices 3 and 4, where a constant DC bias current of both positive and negative magnitude was applied parallel to the a-axis of $TaIrTe_4$. Due to the breaking of mirror symmetry in the ac plane of $TaIrTe_4$, applying current along the a-axis can generate both out-of-plane spin polarization (σ_z) and in-plane spin polarization (σ_{XY}) due to the charge-spin conversion effect[1,26]. This out-of-plane spin polarization can produce unconventional out-of-plane spin-orbit torques (τ_Z), which leads to field-free deterministic switching of any out-of-plane-oriented magnet from the $+m_z$ to $-m_z$, or vice versa[5,8,26,27]. Such unconventional torques can also cause a shift in the AHE loop measured under positive and negative DC bias currents[8,26]. The effective fields (H_{DL}^{Z}) generated by σ_z spin polarization can compete with the out-of-plane external magnetic field. However, these effective fields are of the order of a few millitesla (mT), which is two orders of magnitude smaller than the coercivity of Fe_3GaTe_2 (approximately 200-100 mT). Therefore, to detect such loop shifts, a very large magnitude of DC bias current is required.

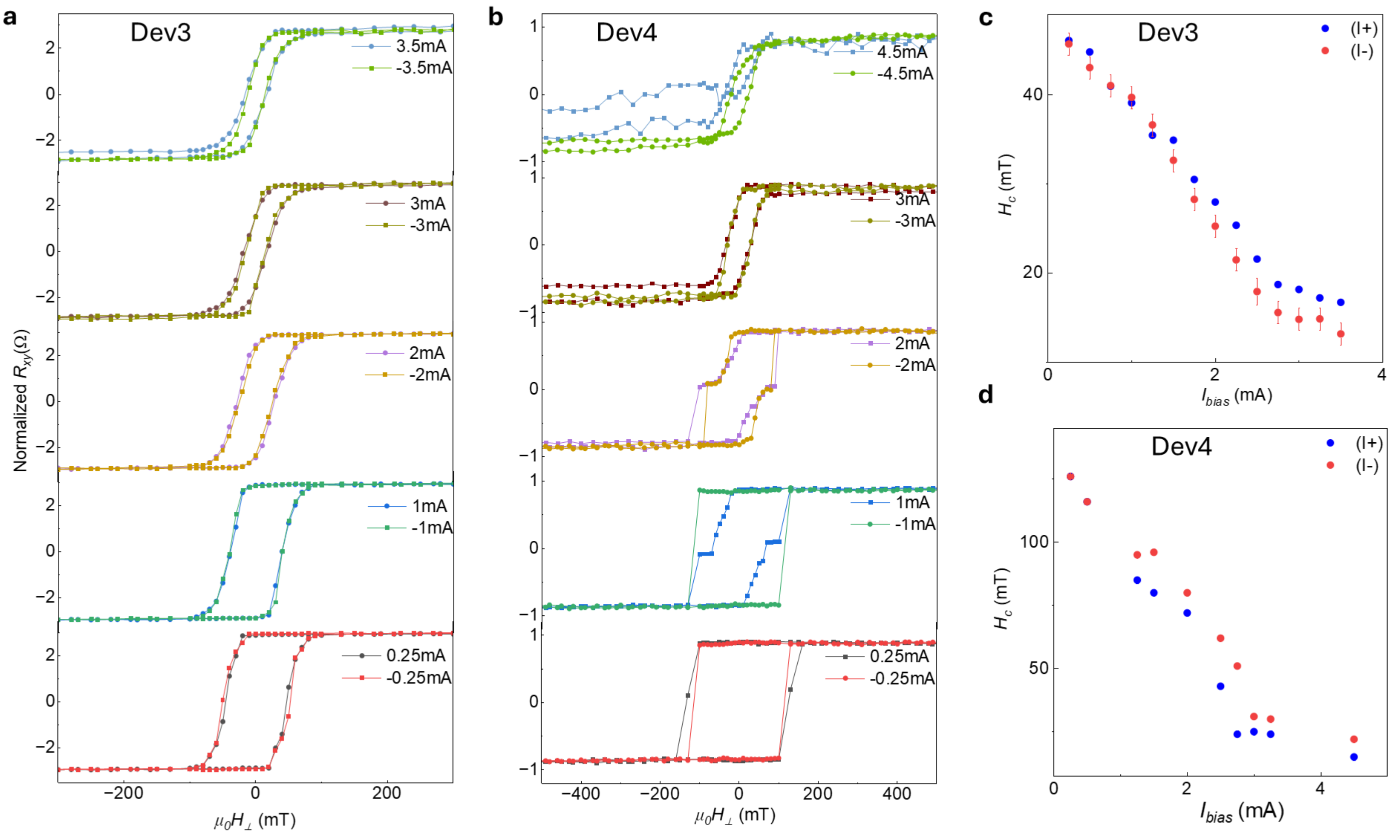

Supplementary Figure S4: Anomalous Hall effect loop shift measurement on Dev3 and Dev4. a,b, *Anomalous Hall loop (R_{xy} vs $\mu_0 H$) under various positive and negative DC bias currents for devices 3 and 4. At lower currents, the loops for positive and negative biases overlap, but a slight shift is observed with increasing current. The AHE loop shifts were found to be approximately 2.3 mT for Dev3 and 4 mT for Dev4 in response to the applied DC bias currents.* ***c,d,*** *Coercivity changes with different magnitudes and polarities of DC bias currents.*

Figure S4 a,b display the AHE loops at different DC bias currents. At a few milliamperes (mA) of positive and negative bias current, the AHE loops overlap. However, as the current increases to |*I*|>3 mA, the AHE loops for positive and negative currents shift by a few millitesla in opposite directions. Figures S4c,d illustrate the decrease in the AHE loop due to the Joule heating effect.

Supplementary Note 5: Current induced change in coercivity and Joule heating effect information

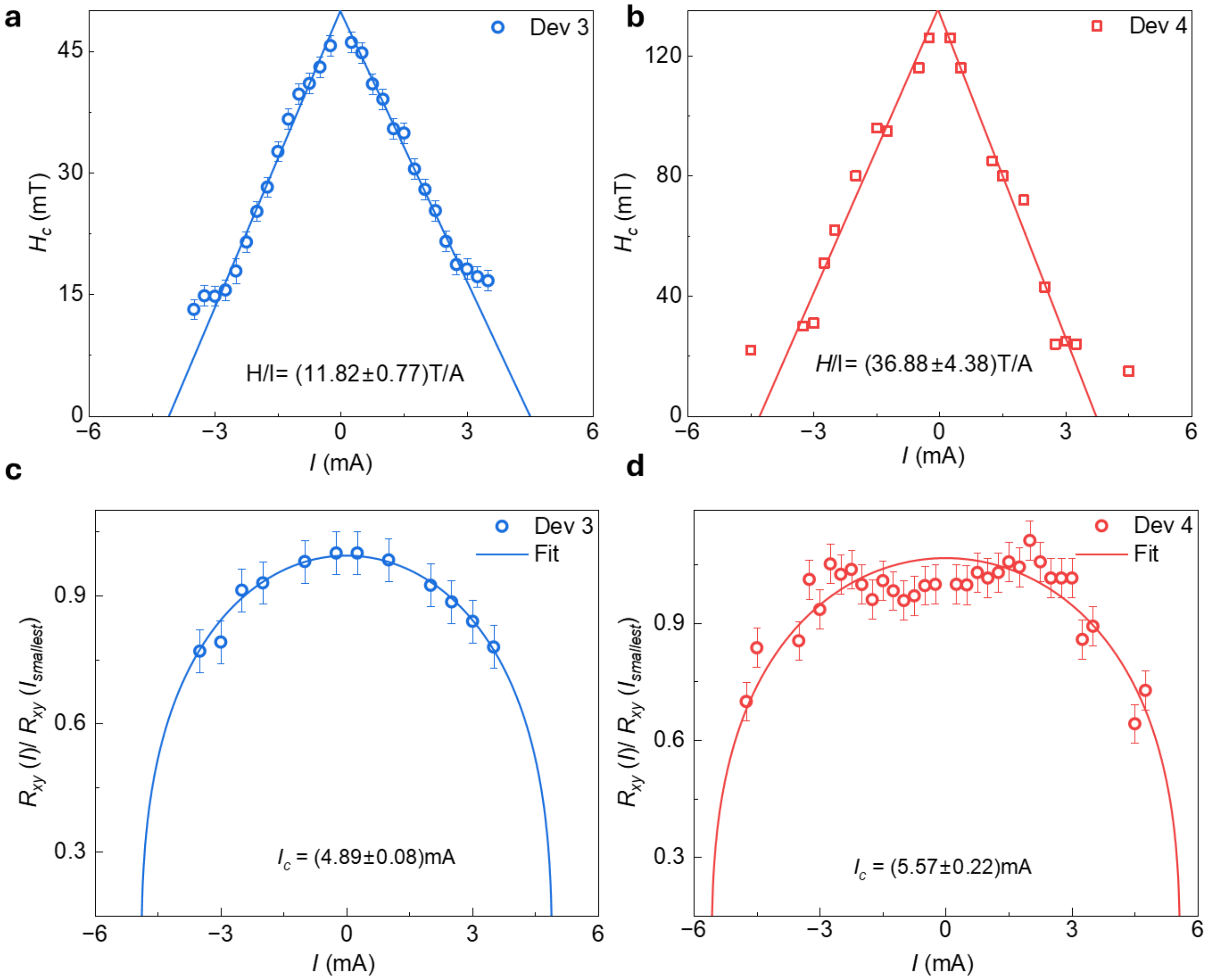

Supplementary Figure S5: Current-induced change in coercivity and anomalous resistance a,b, *Extracted coercivity field (H_c) as a function of applied current (I) for Dev3 and Dev4. Such trend of H_c vs I can be mainly explained by SOT effect and Joule heating effect. The solid line denoted the corresponding linear fit to illustrate the current region up to which H_c change is dominated by SOT. The slope of the curve is shown in the inset of the corresponding figures. Critical current at which free energy barrier vanishes due to SOT and magnetization switching can possibly occur.* ***c,d,*** *Normalized anomalous Hall resistance value as a function of applied current for Dev3 and 4. The curve (R_{xy} vs I) is fitted with equation $R_{xy}(I) = R_{xy}(I_{smallest})\left(1-\left(\frac{I}{I_c}\right)^2\right)^\beta$ analogues to Bloch equation for magnetization vs temperature curve, where exponent β equals to 0.35[9,28–30] and I_c is the critical current at which the device temperature reaches Curie temperature.*

The current-induced spin-orbit torque (H_{SOT}/J) can also reduce the barrier height between the local minima of the free energy of the two-magnetization (±M_s) state other than temperature and external magnetic field[14]. This effect appears as a linear reduction of magnetic coercivity (H_C) with applied current (I). Figures S5a,c illustrate this typical behavior of reduction of H_C with I estimated for Dev3 and Dev4. The trend is fitted with a linear line suggesting SOT-dominated behavior in both devices. However, linear fit deviates to nonlinear around 3mA in Dev 3 and 3.25mA in Dev 4, typically arise due to Joule heating effect in devices. The slope of H_c vs I curve for Dev3 is (11.82±0.77) T/A, equivalent to (2.03±0.06) $\times 10^{-12}\ \mathrm{TA^{-1}m^2}$ and for Dev4 is (36.88±4.38) T/A equivalent to (2.87±0.17) $\times 10^{-12}\ \mathrm{TA^{-1}m^2}$. Similar order of H_{DL}^{z}/J value is also found by second harmonics Hall measurements. The intercept of a linear line is equivalent to the critical current (I_C) at which the free energy

barrier vanished and beyond this current the magnetization current switches[14]. The critical current for Dev3 and Dev4 is around 4 mA (see Fig. S5a,b), roughly matching with the pulse current magnetization switching experiments.

To further deduce the Joule heating information of Dev3 and Dev4, normalized anomalous Hall resistance (i.e., $\mathrm{R_{xy}(I)/R_{xy}(I_{smallest})}$) is plotted with applied current as shown in Fig. S5c,d. The curve (norm R_{xy} vs I) is fitted with equation $\mathrm{R_{xy}(I) = R_{xy}(I_{smallest})}\left(1-\left(\frac{\mathrm{I}}{\mathrm{I_c}}\right)^2\right)^{\beta}$ analogues to Bloch equation for magnetization vs temperature curve where exponent β equal to 0.35[9,28–30] and $\mathrm{I_c}$ is the critical current at which the magnetization device temperature reaches Curie temperature. The device temperature for Dev3 at 3.5mA is roughly around 345 K (*Ms*~0.97×10^5 $\mathrm{Am^{-1}}$)[9], whereas for Dev4, it is around 351 K (*Ms*~0.85×10^5 $\mathrm{Am^{-1}}$)[9] at 4.75 mA current as estimated by comparing fit of Fig. S5c, d and Fig. 2c. The intercept is critical current as shown in Fig. S5c,d is current at which devices temperature reaches Curie temperature. The critical current at which temperature reaches Curie temperature is higher than the critical current needed to switch magnetization state of Fe_3GaTe_2.

Supplementary Note 6. Planar Hall signal of Fe_3GaTe_2 and magnetic field dependent second harmonics behavior of $TaIrTe_4$

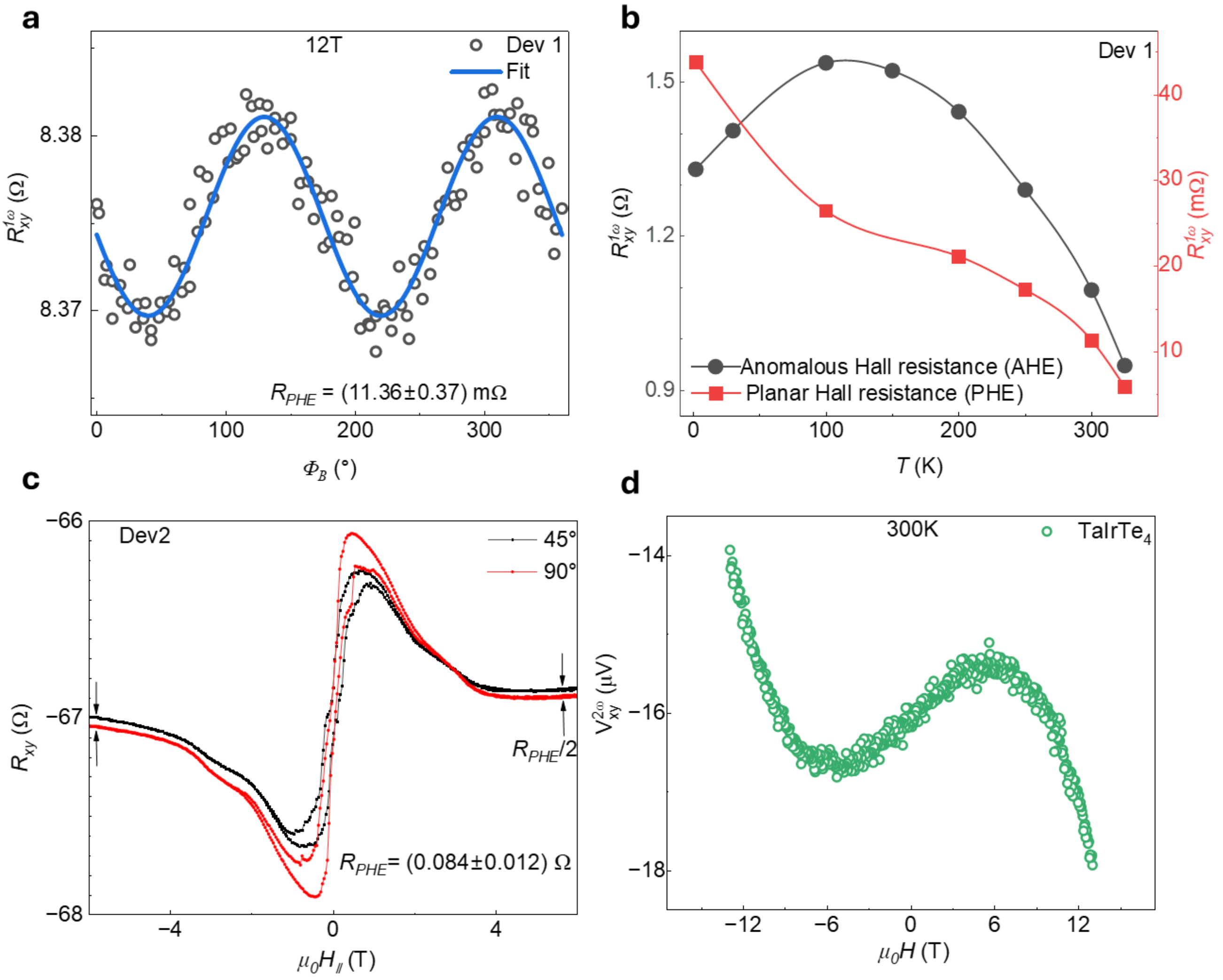

Supplementary Figure S6: Planar Hall effect in Fe_3GaTe_2 and magnetic field dependent second harmonics behavior of $TaIrTe_4$ a. *$R_{xy}^{1\omega}$ as a function of angle (Φ_B) between the in-plane magnetic field (12 T) and applied current measured on the $TaIrTe_4$/Fe_3GaTe_2 (Dev1) heterostructure. The data is fitted with $R_{xy} = R_{PHE} sin\phi_B \cos\phi_B$, yielding R_{PHE} = (11.36±0.37) mΩ.* ***b.*** *Anomalous Hall resistance (AHE) and planar Hall resistance (PHE) as a function of temperature for dev1.* ***c.*** *$R_{xy}^{1\omega}$ as a function of inplane magnetic fields set at 45° and 90° to the injected current measured on Dev2.* ***d.*** *Second harmonic Hall voltage ($V_{xy}^{2\omega}$) as a function of magnetic field measured for only $TaIrTe_4$ device.*

The planar Hall resistance (R_{PHE}) and anomalous Hall resistance (R_{AHE}) are required for calculating spin orbit torque components from the second harmonics Hall measurements on TaIrTe$_4$/Fe$_3$GaTe$_2$ device. The first harmonics Hall resistance ($R_{xy}^{1\omega}$) is measured as a function of angle (Φ_B) between the in-plane constant magnetic field and the applied current. A representative plot of $R_{xy}^{1\omega}$ vs Φ_B for Dev1, measured at 12 T and 300 K, is shown in Fig. S6a. The data is fitted using the equation $R_{xy} = R_{PHE}\sin\phi_B\cos\phi_B$ to extract R_{PHE}, which is determined to be (*11.36±0.37)* mΩ. The temperature dependence of R_{PHE} and R_{AHE} value of Dev1 with temperature is displayed in Fig. S6b. R_{PHE} is two orders of magnitude smaller than R_{AHE} due to strong perpendicular magnetic anisotropy in Fe$_3$GaTe$_2$. Both R_{PHE} and R_{AHE} increase with decreasing temperature, indicating a decrement of thermal randomization of Fe$_3$GaTe$_2$ magnetic moments with temperature. The planar Hall resistance for Dev 2 is estimated using $R_{xy}^{1\omega}$ vs $\mu_0 H_{\parallel}$ measured at 45˚and 90˚to the injected current direction as shown in Fig. S6c. The different between $R_{xy}^{1\omega}$ (45˚)-$R_{xy}^{1\omega}$ (90˚) is equivalent to $R_{PHE}/2$ at large magnetic field. R_{PHE} for Dev2 found out to be ~ (84±12) mΩ which is of the same order as of Dev1.

We used magnetic field and in-plane angular dependent second harmonics Hall (SHH) signal ($V_{xy}^{2\omega}$) to calculate spin-orbit torque components in TaIrTe$_4$/Fe$_3$GaTe$_2$ devices. However, SHH signal can also originate solely from TaIrTe$_4$. The magnetic field dependence of the $V_{xy}^{2\omega}$signal for TaIrTe$_4$ is illustrated in Fig. S6d, showing typical behaviour explained by magneto-chiral anisotropy in topological semimetal system[31]. The $V_{xy}^{2\omega}$ versus $\mu_0 H$ behavior of TaIrTe$_4$ is significantly different than that observed in TaIrTe$_4$/Fe$_3$GaTe$_2$ indicating that the $V_{xy}^{2\omega}$ signal in TaIrTe$_4$/Fe$_3$GaTe$_2$ (see Fig. 4) primarily arises from current-induced spin-orbit torque.

The bilinear magnetoresistance (BMER) in TaIrTe$_4$, as shown in Fig. 1e can also arise due spin dependent inhomogeneities and impurity scattering processes[32]. BMER, due to impurity, is a linear function of current density and its signal amplitude directly correlating to Fermi energy value. However, in the present work, the nonlinear second harmonic signal due to inversion symmetry breaking is a quadratic function of current density (Fig. 1c), and it is minimum at charge neutrality point (i.e., lowest Fermi energy) and changes sign with chemical potential shifts (Fig. 1d).

Supplementary Note 7. Field-like torque components and anomalous Nerst effect in TaIrTe$_4$/Fe$_3$GaTe$_2$

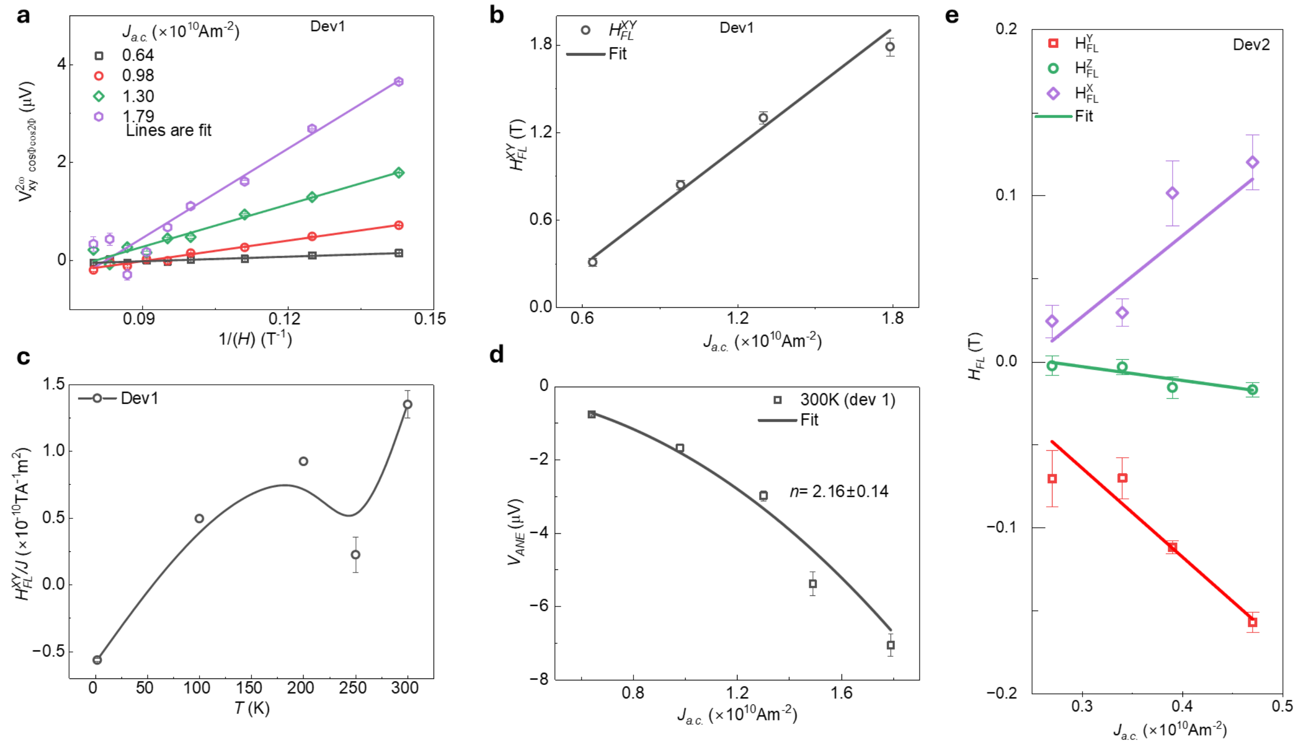

Supplementary Figure S7: Field-like torque and anomalous Nerst effect in TaIrTe₄/Fe₃GaTe₂ a. *$V_{xy}^{2\omega}{}_{\cos\Phi_B\cos2\Phi_B}$ ($\cos\Phi_B\cos2\Phi_B$ dependent in $V_{xy}^{2\omega}$) as a function of 1/H for different current density $J_{a.c.}$. Data is fitted with a linear equation to extract the current-induced SOT field-like component for XY spins (H_{FL}^{XY}) at varying current densities.* ***b.*** *H_{FL}^{XY} as a function of current density. Data is fitted to extract H_{FL}^{XY}/J, which is found to be* (1.35±0.10) $\times 10^{-10}$ $\mathrm{TA^{-1}m^2}$. ***c.*** *Temperature dependence of H_{FL}^{XY}/J for $TaIrTe_4/Fe_3GaTe_2$ heterostructure devices.* ***d.*** *Anomalous Nerst voltage (V_{ANE}) as a function of current density. The data is fitted with power-law equation ($V_{ANE}\alpha J^n$), yielding n = 2.16±0.14. e. $H_{FL}^{X,Y,Z}$ as a function of current density for Dev2. Data is fitted to extract $H_{FL}^{X,Y,Z}/J$.*

To estimate the current-induced effective spin-orbit torques ($\tau_{DL}^X, \tau_{DL}^Y, \tau_{DL}^Z, \tau_{FL}^X, \tau_{FL}^Y, \tau_{FL}^Z$) and field components ($H_{DL}^X, H_{DL}^Y, H_{DL}^Z, H_{FL}^X, H_{FL}^Y\ H_{FL}^Z$) originating from the spin Hall effect in $TaIrTe_4$, which generate both the in-plane and out-of-plane spin polarizations ($\sigma^X, \sigma^Y, \sigma^Z$), we measured the angle and magnetic field dependence on second harmonics signals. The 2nd harmonic transverse voltage generated from these current-induced effective SOT fields ($H_{DL}^{X,Y,Z}, H_{FL}^{X,Y,Z}$) and torques ($\tau_{DL}^{X,Y,Z}, \tau_{FL}^{X,Y,Z}$) in PMA ferromagnets is generally expressed as follows[33,34],

$$V_{xy}^{2\omega} = V_{DL}^Y\cos\Phi_B + V_{DL}^X\sin\Phi_B + V_{DL}^Z\cos2\Phi_B + V_{FL}^Y\cos\Phi_B\cos2\Phi_B + V_{FL}^X\sin\Phi_B\cos2\Phi_B + V_{FL}^Z \quad \text{(Eq. S1)}$$

Where:

$$V_{DL}^Y = H_{DL}^Y\frac{V_{AHE}}{2(H_{ext}-H_K)} + V_{ANE} + V_{ONE}H_{ext} \quad \text{(Eq. S2)}$$

$$V_{DL}^X = H_{DL}^X\frac{V_{AHE}}{2(H_{ext}-H_K)} \quad \text{(Eq. S3)}$$

$$V_{DL}^Z = H_{DL}^Z\frac{V_{PHE}}{(H_{ext})} \quad \text{(Eq. S4)}$$

$$V_{FL}^Y = H_{FL}^Y\frac{V_{PHE}}{(H_{ext})} \quad \text{(Eq. S5)}$$

$$V_{FL}^X = H_{FL}^X\frac{V_{PHE}}{(H_{ext})} \quad \text{(Eq. S6)}$$

$$V_{FL}^Z = H_{FL}^Z\frac{V_{AHE}}{2(H_{ext}-H_K)} + V_{offset} \quad \text{(Eq. S7)}$$

Here, V_{AHE} and V_{PHE} represent the anomalous Hall and planar Hall voltage which can be estimated from 1st harmonic transverse voltage. The anomalous Nerst effect (V_{ANE}), ordinary Nerst effect (V_{ONE}) and V_{offset} denoted contribution from thermal and background voltages.

While fitting the $V_{xy}^{2\omega}$ vs Φ_B using Eq. S1, we found that only one term either V_{FL}^Y or V_{FL}^X, is necessary to fit the data accurately. Consequently, we defined H_{FL}^{XY}. The $V_{xy}^{2\omega}$ vs Φ_B is fit using Eq. S1 and the coefficient $V_{xy}^{2\omega}{}_{\cos\Phi_B\cos2\Phi_B}$ is plotted as a function of 1/H (see Eq. S5). The resulting curve is fitted with a linear equation to determine the slope, corresponding to H_{FL}^{XY}. The H_{FL}^{XY} is subsequently plotted as a function of current density, and the slope of H_{FL}^{XY} versus J curve yielded H_{FL}^{XY}/J, which is found to be (1.35±0.10) × 10^{-10} $\mathrm{TA^{-1}m^2}$. Similarly, the field-like torque components, $H_{FL}^X/J = (0.48 \pm 0.18) \times 10^{-10}\ \mathrm{TA^{-1}m^2}$, $H_{FL}^Y/J = (0.53 \pm 0.087) \times 10^{-10}\ \mathrm{TA^{-1}m^2}$, $H_{FL}^Z/J = (0.083 \pm 0.026) \times 10^{-10}\ \mathrm{TA^{-1}m^2}$ for device 2 extracted from the field dependent harmonic Hall measurements (see Fig. 4) are also lie in the similar range (see Fig. S7e). In both the devices, the field-like torque component is significantly larger than the damping-like torque. However, the value might be overestimated due to Oersted field generated by the applied current, which exhibits similar $\cos\Phi_B\cos2\Phi_B$ angular dependence[35]. Moreover, the second harmonics signal from $TaIrTe_4$ alone, which follow $\cos\Phi_B$ dependence, could contribute to the large observed value of H_{FL}^{XY}/J. Besides that, due to imperfect Hall bar geometry (Fig. S1e), R_{xx} can potential contribute to the second harmonics transverse data, which primarily affect the field-like components[36–38], leading to overestimation in H_{FL}^{XY}/J.

Similarly, the H_{FL}^{XY}/J is estimated at various temperatures, and its temperature dependence is displayed in Fig. S6c. The field-like torque is positive at room temperature and reverses its sign as the temperature decreases. This sign reversal may correlate with the distribution of spin texture near the Fermi level, as depicted in Figs. S12 and S13.

The applied current inducing SOT can generate a temperature gradient due to the Joule heating effect. In the presence of a magnetic field, this temperature gradient generates a transverse Hall voltage. If this voltage arises from the Lorentz force, it is termed as ordinary Nerst effect, typically observed in nonmagnetic systems. Conversely, in magnetic materials, this effect is called anomalous Nerst effect, originating from Berry curvature. Both thermal-induced voltages contribute to the second harmonic transverse voltage, with the signal exhibiting $\cos\Phi_B$ dependence during in-plane magnetic field sweep (see Eq. S2)[39,40]. The ordinary effect can be excluded from the ${V_{xy}^{2\omega}}_{\cos\Phi_B}$ by eliminating any linear field dependence. The intercept of ${V_{xy}^{2\omega}}_{\cos\Phi_B}$ vs 1/(H-H_K) corresponds to anomalous Nerst effect (V_{ANE}). The anomalous Nerst voltage as a function of current density is plotted in Fig. S7d. The curve is fitted with the power law equation $V_{AHE}\ \alpha\ J^n$, where exponent n determined to 2.16 ± 0.14.

Supplementary Note 8. Field dependence and angle dependence second harmonics measurement on TaIrTe₄/Fe₃GaTe₂

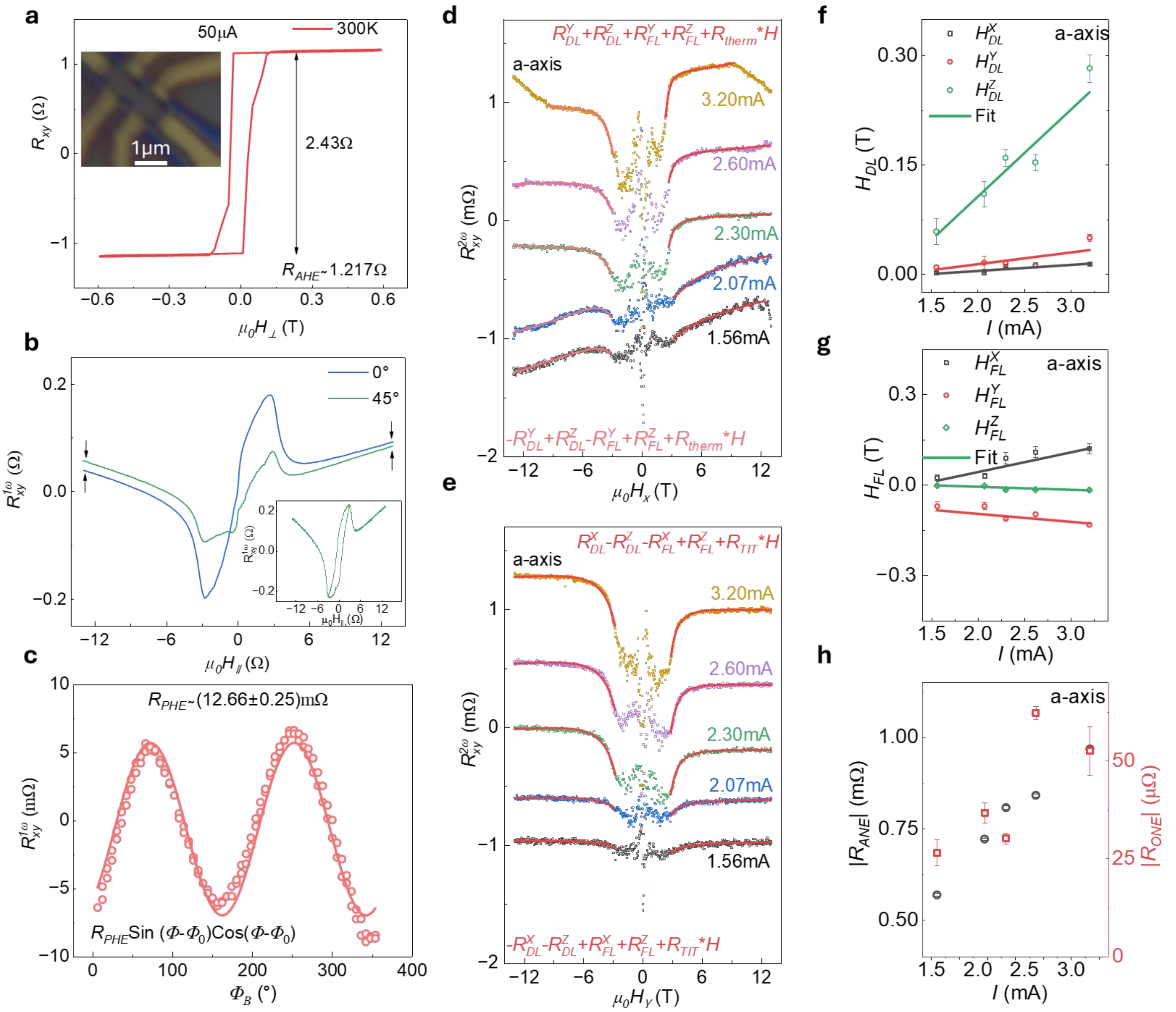

Supplementary Figure S8: Field dependent harmonic Hall measurements in TaIrTe₄/Fe₃GaTe₂ along a-axis of TaIrTe₄
a,b. Transverse resistance (R_{xy}) as a function of out-of-plane and in-plane magnetic field, respectively measurement on Dev5. **c.** $R^{1\omega}_{xy}$ as a function of angle (Φ_B) between the in-plane magnetic field (13 T) and applied current measured on the TaIrTe₄/Fe₃GaTe₂ (Dev5) heterostructure. The data is fitted with $R_{xy} = R_{PHE}\sin(\phi_B - \phi_0)\cos(\phi_B - \phi_0)$, yielding R_{PHE} = (12.66±0.25) mΩ. **d,e,** Dependence of the 2nd harmonic transverse resistance ($R^{2\omega}_{xy}$) on the in-plane magnetic field (H_x and H_y) measured at different magnitude of a.c. current sourced along a-axis of TaIrTe₄. The curve is fit using equations mentioned in inset as simplified from Eq. S1-7. The data are offset in Y to considered background signal at zero field and to avoid overlap. **f,g,h,** Calculated effective damping-like SOT components ($H^{X,Y,Z}_{DL}$), field-like SOT components ($H^{X,Y,Z}_{FL}$) and thermal resistances (R_{ANE} and R_{ONE}) as a function of a.c. current magnitude.

We have measured field and angular dependent harmonic Hall responses on another device (Dev 5) of the TaIrTe₄/Fe₃GaTe₂ heterostructure. The optical image of the device is shown in inset of Fig. S8a. The anomalous Hall resistance, with a of value of 1.21Ω, is estimated from the R_{xy} vs $\mu_0 H_\perp$ plot (Fig. S8a). The transverse resistance as a function of in-plane magnetic field (Fig. S8b) is also measured to estimate magnetic anisotropy field (H_K ~ 2T). The planar Hall resistance is extracted from $R^{1\omega}_{xy}$ as a function of Φ_B (Fig. S8c), and data is fitted using the equation $R_{PHE}\sin(\phi - \phi_0)\cos(\phi - \phi_0)$, where ϕ_0 represents the initial offset angle between the direction of the injected current and the applied in-plane magnetic field. From the fitting, R_{PHE} is estimated to be $(12.66 \pm 0.25) m\Omega$. It should be noted that the R_{PHE} value can also be obtained from $R^{1\omega}_{xy}$ vs $\mu_0 H_\parallel$

measurements performed at fixed Φ_B equal to 0° and 45°. As seen in Fig. S8b, the R_{PHE} values are of the same order as those deduced from Fig. S8c.

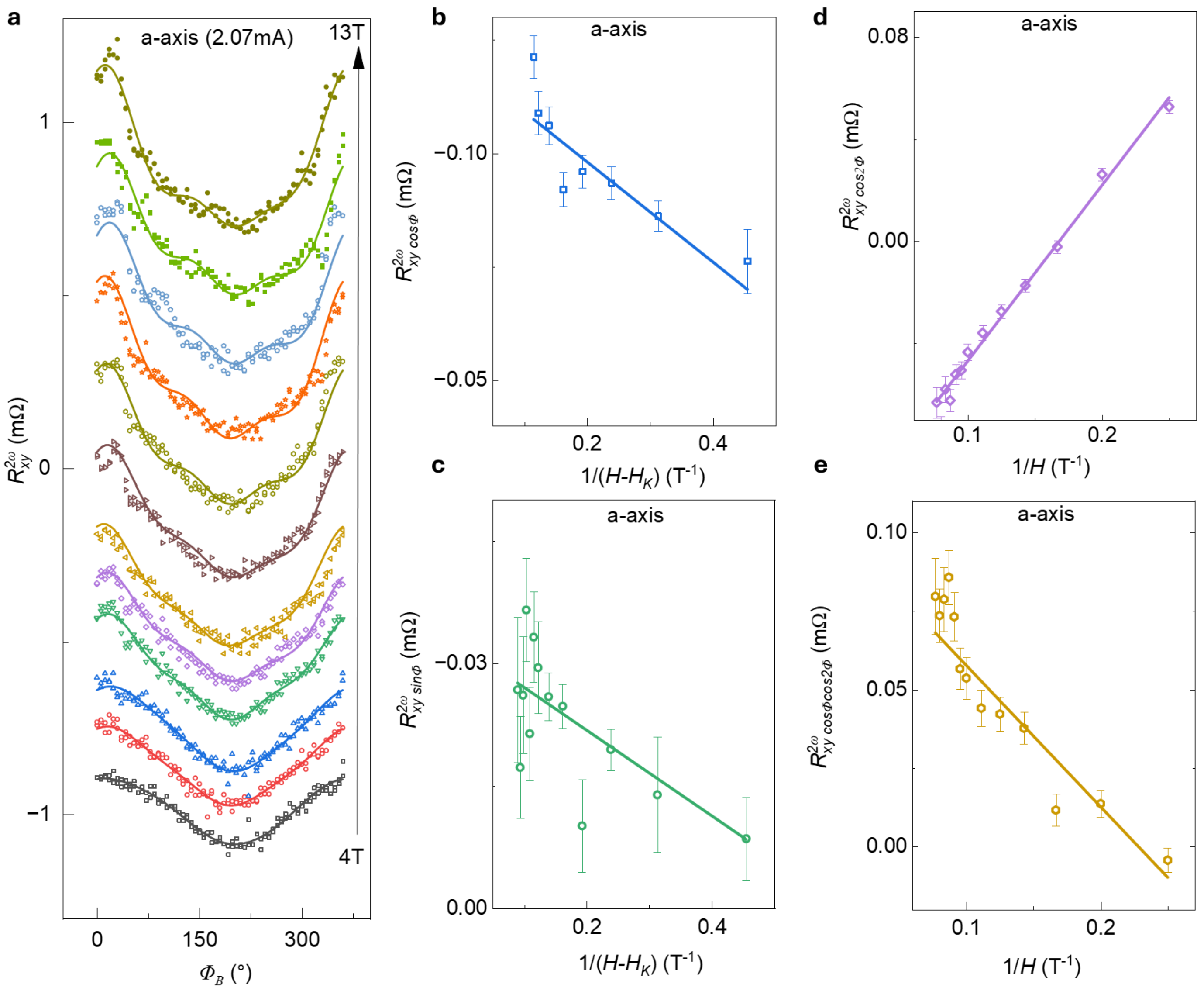

Supplementary Figure S9: Angle dependent harmonic Hall measurements in TaIrTe$_4$/Fe$_3$GaTe$_2$ along a-axis of TaIrTe$_4$ **a,** 2nd harmonic transverse resistance ($R_{xy}^{2\omega}$) as a function of ϕ_B measured at field ranging from 4T to 13T field using 2.07mA a.c. current sourced along a-axis. The data is shifted in Y to avoid overlap. **b,c,d,e.** Coefficient $R_{xy}^{2\omega}{}_{\cos\Phi_B}$, $R_{xy}^{2\omega}{}_{\sin\Phi_B}$, $R_{xy}^{2\omega}{}_{\cos 2\Phi_B}$ and $R_{xy}^{2\omega}{}_{\cos\Phi_B \cos 2\Phi_B}$ as a function of *1/(H-H$_k$)* and *1/H*, respectively. The curve is fitted with linear line to estimate slopes.

To estimate the current-induced effective spin-orbit torques ($\tau_{DL}^X, \tau_{DL}^Y, \tau_{DL}^Z, \tau_{FL}^X, \tau_{FL}^Y, \tau_{FL}^Z$) and field components ($H_{DL}^X, H_{DL}^Y, H_{DL}^Z, H_{FL}^X, H_{FL}^Y\ H_{FL}^Z$) arising from the spin Hall effect in TaIrTe$_4$, we have measured the field dependent second harmonic Hall signal ($R_{xy}^{2\omega}$) along two field direction, H_X, where the magnetic field is parallel to the current axis (which is the a-axis of TaIrTe$_4$ (TIT) in this case) (Fig. S8d), and and H_Y, where the magnetic field is perpendicular to the current axis (also along the a-axis) (Fig. S8e). The $R_{xy}^{2\omega}$ vs H_y data in the positive and negative directions are fitted with the simplified expressions (Eq. S8), derived by setting Φ_B= 90° and Φ_B= 270°, respectively in Eq. S1 and using Eq. S2-S7:

$$V_{xy}^{2\omega} = \pm V_{DL}^{X}\frac{V_{AHE}}{2(|H_y|-H_K)} - H_{DL}^{Z}\frac{V_{PHE}}{|H_y|} \mp H_{FL}^{X}\frac{V_{PHE}}{|H_y|} + H_{FL}^{Z}\frac{V_{AHE}}{2(|H_y|-H_K)} + V_{TIT}|H_y| + V_{offset} \quad \text{(Eq. S8)}$$

Similarly, the $R_{xy}^{2\omega}$ vs H_X data in a positive and negative direction are fitted with expression Eq. S9 derived by putting Φ_B= 90° and Φ_B= 270, respectively in Eq. S1 and using Eq. S2-S7:

$$V_{xy}^{2\omega} = \pm V_{DL}^{Y}\frac{V_{AHE}}{2(|H_x|-H_K)} + H_{DL}^{Z}\frac{V_{PHE}}{|H_x|} \pm H_{FL}^{Y}\frac{V_{PHE}}{|H_x|} + H_{FL}^{Z}\frac{V_{AHE}}{2(|H_x|-H_K)} + V_{ONE}|H_x| + V_{ANE/offset} \quad \text{(Eq. S9)}$$

From the fitting and analysis, the damping like ($H_{DL}^{X,Y,Z}$) and field-like ($H_{DL}^{X,Y,Z}$) SOT field components are estimated. Their values, determined at various current amplitudes, are shown in Fig. S8f,g. The calculated data is further fitted with linear line to estimate $\frac{H_{DL}^{X}}{I} = 8.37 \pm 2.92\ T/A$, $\frac{H_{DL}^{Y}}{I} = 16.24 \pm 8.51 T/A$, $\frac{H_{DL}^{Z}}{I} = 119.71 \pm 28.23 T/A$, $\frac{H_{FL}^{X}}{I} = (65.05 \pm 19.34)\ T/A$, $\frac{H_{FL}^{Y}}{I} = (26.37 \pm 19.55) T/A$ and $\frac{H_{FL}^{Z}}{I} = (9.87 \pm 3.25) T/A$. From this analysis, we conclude that the unconventional out of plane damping like torque is significantly larger than in-plane damping like components in $TaIrTe_4/Fe_3GaTe_2$ bilayer.

It is to be noted here that $H_{DL/FL}$ vs current (I) plots exhibit a finite nonzero intercept along with a slope. However, theoretical model for spin orbit torque predicts that $H_{DL/FL}$ vs I behavior should ideally follow a line passing through the origin. The deviation from this ideal behavior can be explained by a finite contribution from the second harmonic signal originating from $TaIrTe_4$ itself, which exhibits a nonlinear dependence with current, as shown in Fig. 1 and Figs. S2c,d.

Furthermore, the thermal contributions R_{ONE} and R_{ANE}, as shown in Fig. S8h, are also estimated by fitting the $R_{xy}^{2\omega}$ vs H_X data, by incorporating $R_{ANE} + R_{ONE}H_{ext}$ term while fitting the data (see equation S8). Moreover, it appears from the $R_{xy}^{2\omega}$ vs H_x data there is an additional contribution of the form $R*H$ having an opposite slope to $R_{ONE}H_{ext}$. This feature is clearly visible in the $R_{xy}^{2\omega}$ vs H_X data measured at 3.20mA. Such a contribution might originate from the second harmonic signal of $TaIrTe_4$ itself, potentially leading to an underestimation of the R_{ONE} and R_{ANE} values. Moreover, this contribution can also be incorporated by including a term of the form $R_{offset} + R_{TIT}H_{ext}$ while fitting the $R_{xy}^{2\omega}$ vs H_y data. We have also performed a similar field dependent second harmonic measurements by injecting a.c. current along b-axis as shown in Fig.S10c,d. In this configuration, both $R_{xy}^{2\omega}$ vs H_x and H_y data shows a dominant monotonically increasing H dependence rather than a $1/H$ or $1/H$-H_k behavior, contrary to the data obtained with current along the low symmetric crystallographic axis (i.e., a-axis). This indicates that the second harmonic signals primarily originate from $TaIrTe_4$ (see Fig. S6d) and associated thermal effects, with only minor contributions from SOT in the b-axis configuration.

Another important point to conclude from Eq. S8 and Eq. S9 is that, in the absence of any unconventional SOT components (i.e., $H_{DL/FL}^{Z}$), the $V_{xy}^{2\omega}$ vs H_{ext} data should be completely antisymmetric with respect to positive and negative magnetic fields. However, in our measurements of $V_{xy}^{2\omega}$ vs H_{ext} performed on both Dev2 (see Fig. 4) and Dev5 (see Fig. S8), where current is injected along the a-axis of $TaIrTe_4$, a finite symmetric component is observed. This indicates a significant contribution from the unconventional SOT component. In contrast, the $V_{xy}^{2\omega}$ vs H_{ext} data for Dev5, when measured by sourcing current along the b-axis of $TaIrTe_4$, appears almost completely antisymmetric, suggesting absence of any $H_{DL/FL}^{Z}$ contribution.

Similarly, angular dependent harmonic Hall measurement, shown in Fig. S9a,b, is also performed on Dev5 to estimate the SOT components. $R_{xy}^{2\omega}$ as a function of ϕ_B is measured at various field strengths using an a.c. current source along both the a- and b-axis of $TaIrTe_4$ (see Figs. S9a,b and Figs. S10a,b). Notably, the $R_{xy}^{2\omega}$ vs ϕ_B data acquired using current sourced along the a-axis (Fig. S9a) cannot be fitted using Eq. 2, which includes only conventional (XY) damping-like and field-like torque terms. Instead, additional unconventional torque components (Z) (see Eq. 1 or Eq. S1) are required to accurately fitted the data. In contrast, when current is injected along the b-axis, the $R_{xy}^{2\omega}$ vs ϕ_B data convincingly fitted with Eq. 2, indicating a strong out-of-plane spin polarization and the presence of unconventional SOT components (e.g., τ_{DL}^{Z}) that appear only when current flows along the low-symmetry axis of $TaIrTe_4$. From the fits of $R_{xy}^{2\omega}$ vs ϕ_B curve at various magnetic field strengths (Fig. S9b), the coefficient ${R_{xy}^{2\omega}}_{\cos\Phi_B}$, ${R_{xy}^{2\omega}}_{\sin\Phi_B}$, ${R_{xy}^{2\omega}}_{\cos 2\Phi_B}$ and ${R_{xy}^{2\omega}}_{\cos\Phi_B \cos 2\Phi_B}$ are estimated and plotted as a function of $1/(H$-$H_k)$ and $1/H$, respectively, in Fig. S9b-e. From the slope of these plots, and using the estimated R_{AHE} and R_{PHE} values, the SOT fields $H_{DL}^{X}, H_{DL}^{Y}, H_{DL}^{Z}$ and H_{FL}^{Y} are estimated to be (0.103±0.029)mT, (0.017±0.0045)mT, (34.40±1.07)mT and (22.40±2.34)mT, respectively further confirming that the out-of-plane damping-like torque component is significantly larger than the in-plane components. Again, ideally, the $R_{xy}^{2\omega}$ vs

1/H or *1/(H-H_k)* curves pass through the origin. The observed finite intercepts can be attributed to thermal effects and the intrinsic second harmonic contribution from $TaIrTe_4$, both of which increase linearly with field. These contributions can be subtracted by fitting high field data (e.g., *H*=10-13) with a linear function of the form *s.H+i* and then removing *s.H+i* from the $R_{xy}^{2\omega}$ vs *H* data, where with *s* and *i* are extracted slope and intercept of linear fit, respectively.

In addition, it can be also be observed from the angular-dependent harmonic Hall data on Dev2 and Dev5 (see Fig. 3 and Figs. S10a,b) that $R_{xy}^{2\omega}(\phi_B) \neq R_{xy}^{2\omega}(\phi_B \pm 180^{\circ})$ when the current is sourced along the a-axis of $TaIrTe_4$, suggesting the finite influence of SOT field components that contribute symmetrically under $\phi_B \pm 180^{\circ}$ rotation. In contrast, when the current is sourced along the b-axis, the $R_{xy}^{2\omega}$ vs ϕ_B data illustrating the behavior $R_{xy}^{2\omega}(\phi_B) = R_{xy}^{2\omega}(\phi_B \pm 180^{\circ})$, providing evidence for the absence of any symmetric contribution from out of plane (Z) SOT components.

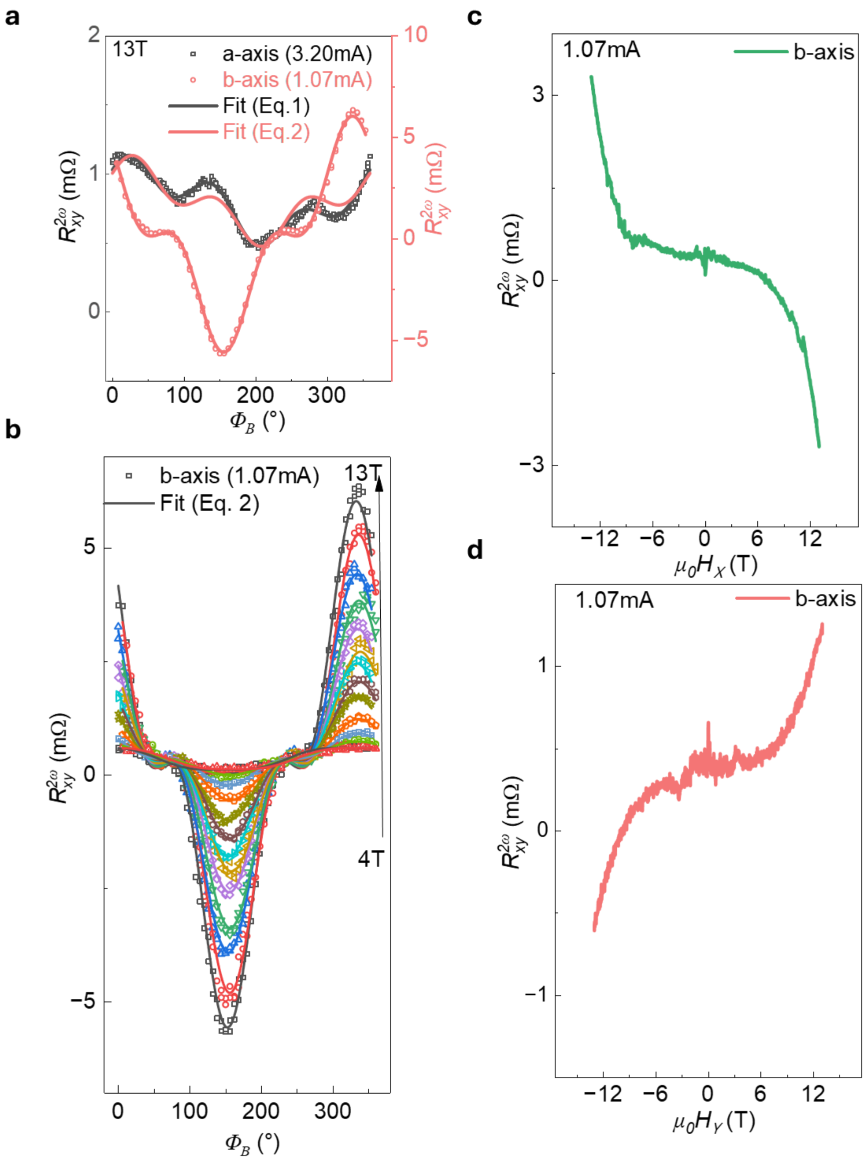

Supplementary Figure S10: Angle and field dependent harmonic Hall measurements in $TaIrTe_4/Fe_3GaTe_2$ along b-axis of $TaIrTe_4$ a, 2nd harmonic transverse resistance ($R_{xy}^{2\omega}$) as a function of ϕ_B measured at 13T when current injected along a and b-axis. The curves are fitted with Eq 1 and 2 describe in main text. **b,c,** 2nd harmonic transverse resistance ($R_{xy}^{2\omega}$) as a function of ϕ_B measured at field ranging from 4T to 13T field using 1.07mA a.c. current sourced along b-axis. **c,d,** $R_{xy}^{2\omega}$ as a function of H_x *and* H_y measured at ~1mA a.c. current source along b-axis of $TaIrTe_4$.

Supplementary Note 9: Space group of bulk $TaIrTe_4$

The relaxed structure of bulk $TaIrTe_4$ was analyzed using the spglibcode[41]. The tolerance parameter (symprec) that determines whether two points occupy the same position was set to 10^{-3}, 10^{-2}, and 10^{-1}, yielding space group (SG) 1, SG 6, and SG 31, respectively. Note that it always implies a minor deviation from SG 31.

The cell parameters and relaxed coordinates are given below:

CELL PARAMETERS (angstrom)		
3.7700000000	0.0000000000	0.0000000000
0.0000000000	12.4210000000	0.0000000000
0.0000000000	0.0000000000	13.1840000000

ATOMIC POSITIONS (angstrom)			
Ir	0.0141414285	6.8219068100	0.3939240715
Ta	0.0142475542	0.8294283236	0.4520497305
Te	0.0142511931	4.4354950648	1.6638122163
Te	0.0141713175	10.7035667918	1.8330590116
Te	1.8992737553	7.4341869275	2.2629711160
Te	1.8989504444	1.4594535089	2.3831905236
Te	1.8998539983	4.0700613395	4.9224056411
Te	1.8990384973	10.1097743022	4.9926354864
Te	0.0134052180	0.8857199404	5.5098556699
Te	0.0140767007	7.0899525333	5.5733980420
Ta	0.0146738082	3.4289284594	6.8542217301
Ir	0.0139559831	9.4764372657	6.8608716585
Ir	1.8992431832	5.8215488459	6.9780759647
Ta	1.8989083366	11.8145444157	7.0458569050
Te	1.8990149178	8.2039639638	8.2572699737
Te	1.8998525481	1.9430435936	8.4199550882
Te	0.0140406486	5.2104768558	8.8456983327
Te	0.0135208496	11.1835812327	8.9782253671
Te	0.0143431103	8.5744940946	11.5224265995
Te	0.0142481238	2.5307932376	11.5808446556
Te	1.8988865875	11.7539205127	12.1059280872
Te	1.8992493938	5.5569890919	12.1699376816
Ta	1.8995261838	9.2131978302	13.4548678497
Ir	1.8994590944	3.1664527045	13.4491500312

Supplementary Note 10: Calculated Fermi surfaces of bulk $TaIrTe_4$

The calculated electronic structure of bulk $TaIrTe_4$ is in good agreement with the previous first-principles simulations and experimental results[42]. Figure S11 shows the calculated Fermi surfaces corresponding to $E = E_F$. The four Fermi sheets displayed in (a) are color-coded according to the band number, while panel (b) shows individual bands with the color indicating the corresponding k-resolved spin Berry curvature Ω_{zx}^{z} responsible for the unconventional spin Hall effect. Our analysis suggests that at the Fermi level, the main contribution comes from the weakly k_y-dispersive band denoted as II. Varying the chemical potential changes the contributions from different bands, even though unconventional SHC remains nearly constant close to E_F.

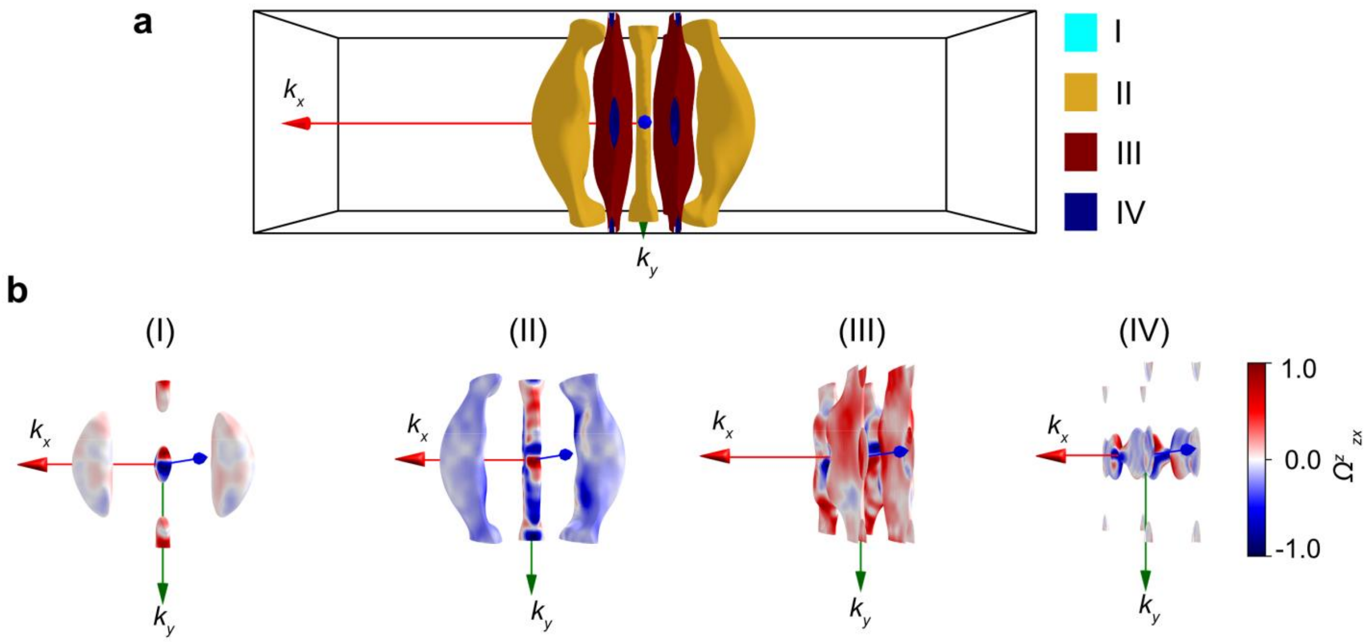

Supplementary Figure S11: ***(a)*** *Fermi surface at E = 0.0 eV consisting of four sheets.* ***(b)*** *Spin Berry curvature distribution* $\Omega_{zx}^{z}(k)$ *for all four bands at E = 0.0 eV. The maximum/minimum values are normalized to -1/1. The sum of the spin Berry curvatures coming from these bands at E = 0.0 eV vanishes except for the band labeled (II) in (b), where it yields a net negative value; hence, mainly this contributes to unconventional SHC at E = 0.0 eV. The sizes of the Fermi sheets in (b) are rescaled to better visualize* Ω_{zx}^{z}.

Supplementary Note 11: Spin texture and Rashba-Edelstein effect in bulk $TaIrTe_4$.

We additionally analyzed the spin textures and Rashba-Edelstein effect in bulk $TaIrTe_4$. Figure S12 shows the spin-resolved band structures along the high-symmetry lines, which reveal mostly in-plane components, similar to other transition metal dichalcogenides T_d-WTe_2 and T_d-$MoTe_2$[43,44]. The S_z component of the spin texture is still present in some regions that do not coincide with the high-symmetry lines. It is exemplified in Fig. S13, where the Fermi surface – corresponding to band II – is color-coded with the S_z component of the spin texture (c). We additionally display the spin-splitting of this Fermi sheet (b); the splitting turns out to be large across the band, suggesting that the S_z component of spin texture could be detected at room temperature.

The calculated bulk Rashba-Edelstein effect (REE) reveals two nonzero components, χ_{xy} and χ_{yx}, with values overall comparable with the previously reported results for T_d-WTe_2[45]. However, in contrast to the latter, the

values of the REE response tensor near the Fermi level are close to zero. This indicates that the Rashba-Edelstein effect may not play a major role in the charge-to-spin conversion in bulk $TaIrTe_4$.

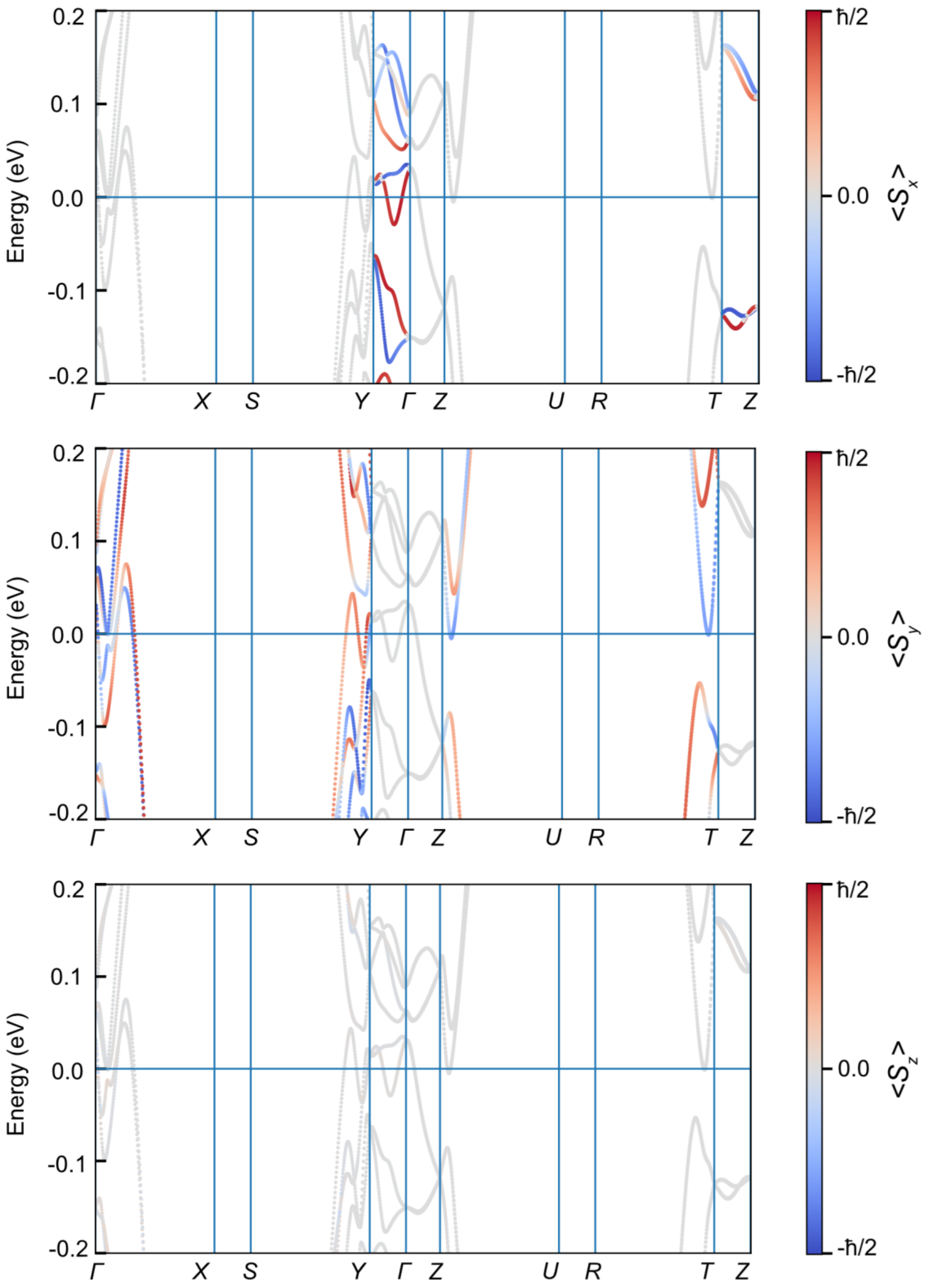

***Supplementary Figure S12**: Calculated spin-resolved band structure along the high-symmetry lines defined in Fig.9 in the main text. Three different panels represent S_x, S_y, and S_z components of the spin texture.*

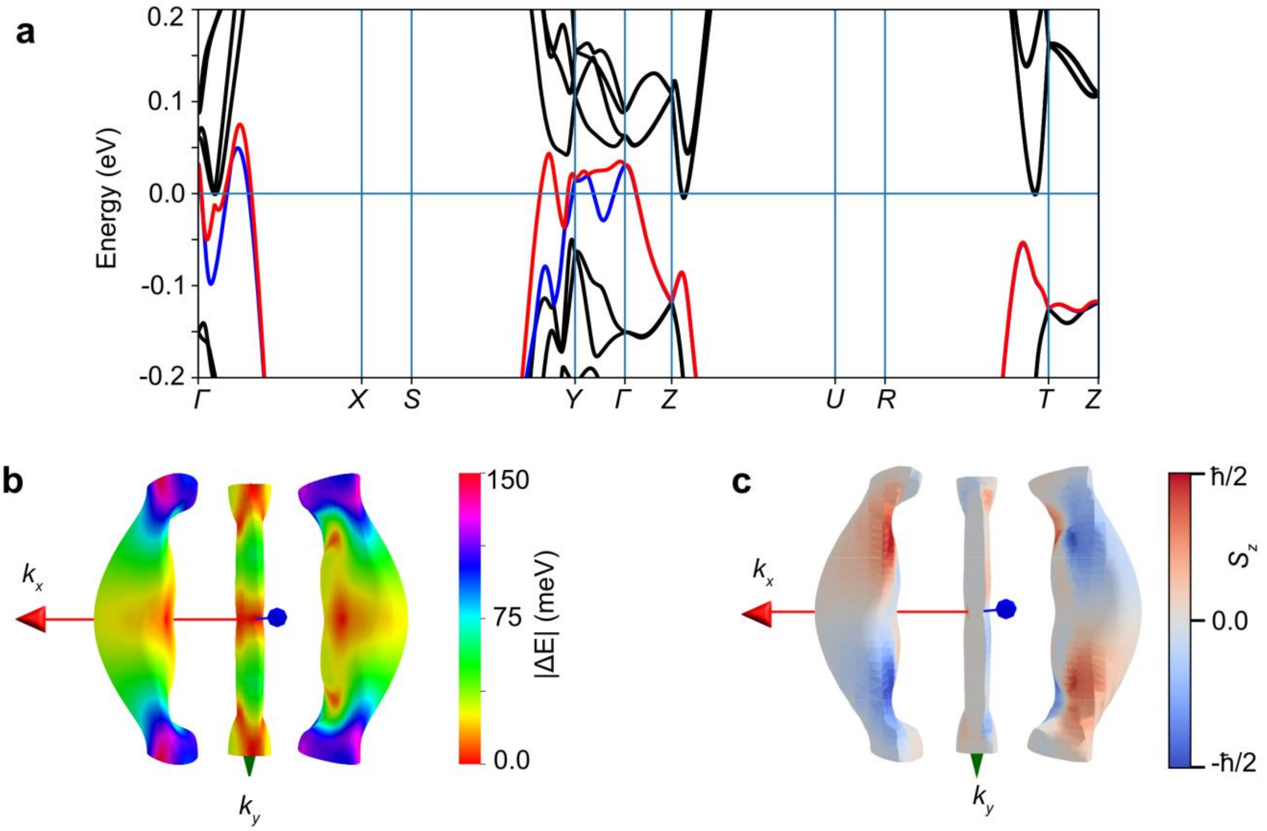

Supplementary Figure S13: (a) *Calculated band structure of bulk $TaIrTe_4$ as in Fig. 6 in the main text. The band denoted in red is a dominant contribution to the unconventional spin Hall conductivity at the Fermi level, and the band beneath is marked as blue.* ***(b)*** *The constant energy surface (Fermi surface) corresponds to the red band at $E=E_F$. The color coding denotes the value of spin splitting, i.e. the difference between the red and blue bands in (a).* ***(c)*** *The same as (b) but with color coding corresponding to the S_z component of the spin texture.*

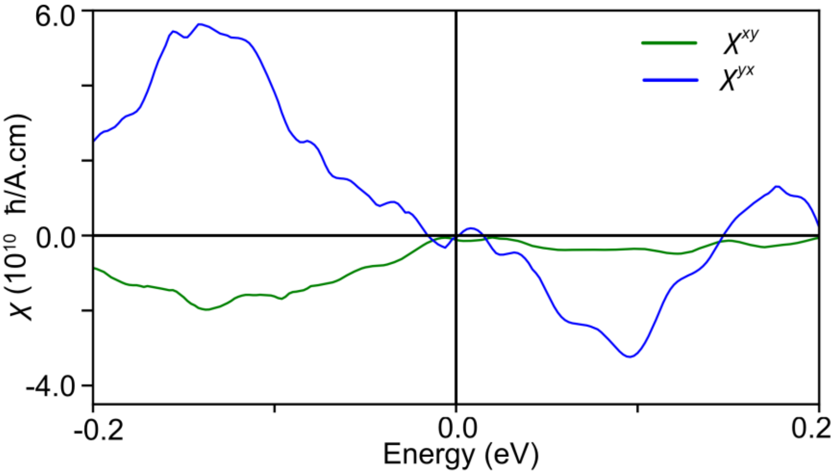

Supplementary Figure S14*: Calculated Rashba-Edelstein response tensor vs chemical potential. For tensor elements χ_{xy} (χ_{yx}), the charge current along the y-axis (x-axis) induces spin density with spins aligned along the x-axis (y-axis). The other components are negligible, which implies that the out-of-plane spin polarization cannot be generated by the Rashba-Edelstein effect in bulk $TaIrTe_4$.*

Supplementary Note 12: Comparison of spin orbit torque parameters with state-of-the-art results

Supplementary Table 1. Summary of spin orbit torque parameters such as SOT efficiency (unconventional SOT efficiency if z-spin is present) (ε_{SOT}), switching current density (J_{sw}), charge conductivity of spin orbit material (σ_C), out-of-plane spin Hall conductivity (σ^Z_{SH}), in-plane spin Hall conductivity (σ^{XY}_{SH}) and switching power density (P) in state-of-the-art results. The thickness that are mentioned in brackets are in nanometers.

System	ε_{SOT}	J_{sw} ($\times 10^{10}$Am^{-2})	$\sigma_C \times 10^5$ $(\Omega m)^{-1}$	σ^Z_{SH} ($\times 10^5$) ħ/2e$(\Omega m)^{-1}$	σ^{XY}_{SH} ($\times 10^5$) ħ/2e$(\Omega m)^{-1}$	P ($\times 10^{15}$) ħ/2e$(\Omega m)^{-1}$	Ref.
Mn_3Sn(7)/Cu(1)/[Ni(0.4)/Co(0.2)]$_3$)/Cu(1)	0.067	4.60	2.72	0.183	0.602	7.77	46
Pt(6)/Fe_3GaTe_2(20)	0.28	13					9
$(Bi,Sb)_2(Se,Te)_3$(32)/Fe_3GaTe_2(9)	5.77	0.90					47
Pt(6)/Fe_3GaTe_2(58)	0.093	1.69	12.50		1.163	0.228	7
Bi_2Te_3(8)/Fe_3GaTe_2(4)	2.69	2.20					48
$(Bi,Sb)_2Te_3$(6)/Ti(2)/CoFeB(1.4)	2.500	0.52	0.18		0.460	1.47	33
$MoTe_2$(0.7)/$Ni_{80}Fe_{20}$(6)	0.032		0.18		0.0058		49
$PtTe_2$(5)/$Ni_{80}Fe_{20}$(10)	0.5-0.15		30-300		0.2-2		50
WTe_2(2.6)/Ti(1.5)/CoFeB(1.2)	0.020	3.25	1.50	0.358	0.205	7.04	51
$TaIrTe_4$(30)/Ti(2)/CoFeB(0.9)	0.043	7.65	4.77	0.206	0.540	12.27	26
$TaIrTe_4$(10)/Ti(2)/CoFeB(1.2)	0.050	2.35	4.91	0.247	0.308	1.173	4
WTe_2(10)/Ti(2)/CoFeB(1.2)	0.013	1.30	0.24	0.032	0.038	6.93	4
$PtTe_2$(2)/WTe_2(6)/Ti(2)/CoFeB(0.9)	0.034	2.25	0.89	0.250	2.350	5.68	52
$TaIrTe_4$(20-120)/$Ni_{80}Fe_{20}$(5-6)	0.11		0.19-3.66	0.405	0.143		1
WTe_2(10-15)/Fe_3GeTe_2(<10) (150K)	4.60	3.90	2.25	10.35		6.76	53
$TaIrTe_4$(10.3)/Fe_3GaTe_2(4.8)	0.37	2.56	6.75	2.60	0.2	0.97	11
$TaIrTe_4$(52-78)/Fe_3GaTe_2 (46-50)	1.24-1.76	1.81-4.15	9.40	11.65-16.50	0.582	0.348-1.832	This work

References

1. Bainsla, L. *et al.* Large out-of-plane spin–orbit torque in topological Weyl semimetal TaIrTe4. *Nat. Commun.* **15**, 4649 (2024).
2. Liu, Y. *et al.* Raman Signatures of Broken Inversion Symmetry and In-Plane Anisotropy in Type-II Weyl Semimetal Candidate TaIrTe4. *Adv. Mater.* **30**, 1706402 (2018).
3. Kumar, D. *et al.* Room-temperature nonlinear Hall effect and wireless radiofrequency rectification in Weyl semimetal TaIrTe4. *Nat. Nanotechnol.* **16**, 421–425 (2021).
4. Zhang, Y. *et al.* Room temperature field-free switching of perpendicular magnetization through spin-orbit torque originating from low-symmetry type II Weyl semimetal. *Sci. Adv.* **9**, eadg9819 (2023).
5. Kajale, S. N., Nguyen, T., Hung, N. T., Li, M. & Sarkar, D. Field-free deterministic switching of all–van der Waals spin-orbit torque system above room temperature. *Sci. Adv.* **10**, eadk8669 (2024).

6. Highly Efficient Room-Temperature Spin-Orbit-Torque Switching in a Van der Waals Heterostructure of Topological Insulator and Ferromagnet. *Advanced Science* vol. 11 2400893 (2024).

7. Kajale, S. N. *et al.* Current-induced switching of a van der Waals ferromagnet at room temperature. *Nat. Commun.* **15**, 1485 (2024).

8. Kao, I.-H. *et al.* Deterministic switching of a perpendicularly polarized magnet using unconventional spin–orbit torques in WTe2. *Nat. Mater.* **21**, 1029–1034 (2022).

9. Li, W. *et al.* Room-Temperature van der Waals Ferromagnet Switching by Spin-Orbit Torques. *Adv. Mater.* **35**, 2303688 (2023).

10. Li, D. *et al.* Room-temperature van der Waals magnetoresistive memories with data writing by orbital current in the Weyl semimetal TaIrT e 4. *Phys. Rev. B* **110**, 035423 (2024).

11. Z hang, Y. *et al.* Robust Field-Free Switching Using Large Unconventional Spin-Orbit Torque in an All-Van der Waals Heterostructure. *Adv. Mater.* **36**, 2406464 (2024).

12. Zhang, G. *et al.* Room-temperature Highly-Tunable Coercivity and Highly-Efficient Multi-States Magnetization Switching by Small Current in Single 2D Ferromagnet Fe_3 $GaTe_2$. *ACS Mater. Lett.* **6**, 482–488 (2024).

13. Deng, Y. *et al.* Room-Temperature Highly Efficient Nonvolatile Magnetization Switching by Current in van der Waals Fe_3 $GaTe_2$ Devices. *Nano Lett.* **24**, 9302–9310 (2024).

14. Zhang, K. *et al.* Gigantic Current Control of Coercive Field and Magnetic Memory Based on Nanometer-Thin Ferromagnetic van der Waals Fe_3 $GeTe_2$. *Adv. Mater.* **33**, 2004110 (2021).

15. Saunderson, T. G., Go, D., Blügel, S., Kläui, M. & Mokrousov, Y. Hidden interplay of current-induced spin and orbital torques in bulk Fe 3 GeTe 2. *Phys. Rev. Res.* **4**, L042022 (2022).

16. Johansen, Ø., Risinggård, V., Sudbø, A., Linder, J. & Brataas, A. Current Control of Magnetism in Two-Dimensional \mathrmFe_3\mathrmGeTe_2. *Phys Rev Lett* **122**, 217203 (2019).

17. Zhang, K. *et al.* Highly Efficient Nonvolatile Magnetization Switching and Multi-Level States by Current in Single Van der Waals Topological Ferromagnet Fe_3 $GeTe_2$. *Adv. Funct. Mater.* **31**, 2105992 (2021).

18. Zhang, K.-X. *et al.* Broken Inversion Symmetry in Van Der Waals Topological Ferromagnetic Metal Iron Germanium Telluride. *Adv. Mater.* **36**, 2312824 (2024).

19. Martin, F. *et al.* Strong bulk spin–orbit torques quantified in the van der Waals ferromagnet $Fe_3 GeTe_2$. *Mater. Res. Lett.* **11**, 84–89 (2023).

20. Robertson, I. O. *et al.* Imaging current control of magnetization in Fe3GeTe2 with a widefield nitrogen-vacancy microscope. *2D Mater.* **10**, 015023 (2022).

21. Zhang, H. *et al.* Room-Temperature, Current-Induced Magnetization Self-Switching in A Van Der Waals Ferromagnet. *Adv. Mater.* **36**, 2308555 (2024).

22. Neumann, L. & Meinert, M. Influence of the Hall-bar geometry on harmonic Hall voltage measurements of spin-orbit torques. *AIP Adv.* **8**, 095320 (2018).

23. Chen, G. X., Cao, R. X., Zholud, A. & Urazhdin, S. Measurement of the Hall effect at nanoscale with three probes. *Rev. Sci. Instrum.* **89**, 083904 (2018).

24. Li, D. *et al.* Roles of Joule heating and spin-orbit torques in the direct current induced magnetization reversal. *Sci. Rep.* **8**, 12959 (2018).

25. Zhu, L. Switching of Perpendicular Magnetization by Spin–Orbit Torque. *Adv. Mater.* **35**, 2300853 (2023).

26. Liu, Y. *et al.* Field-free switching of perpendicular magnetization at room temperature using out-of-plane spins from TaIrTe4. *Nat. Electron.* **6**, 732–738 (2023).

27. MacNeill, D. *et al.* Control of spin–orbit torques through crystal symmetry in WTe2/ferromagnet bilayers. *Nat. Phys.* **13**, 300–305 (2017).

28. Herring, C. & Kittel, C. On the Theory of Spin Waves in Ferromagnetic Media. *Phys. Rev.* **81**, 869–880 (1951).

29. Sato, K. *et al.* Weak itinerant ferromagnetism in Heusler-type \textFe_2\textVAl_0.95. *Phys Rev B* **82**, 104408 (2010).

30. Mishra, V. *et al.* Investigation of spin gapless semiconducting behaviour in quaternary CoFeMnSi Heusler alloy thin films on Si (1 0 0). *J. Magn. Magn. Mater.* **547**, 168837 (2022).

31. Wang, Y. *et al.* Gigantic Magnetochiral Anisotropy in the Topological Semimetal ZrTe 5. *Phys. Rev. Lett.* **128**, 176602 (2022).

32. Dyrdał, A., Barnaś, J. & Fert, A. Spin-Momentum-Locking Inhomogeneities as a Source of Bilinear Magnetoresistance in Topological Insulators. *Phys. Rev. Lett.* **124**, 046802 (2020).

33. Wu, H. *et al.* Room-Temperature Spin-Orbit Torque from Topological Surface States. *Phys. Rev. Lett.* **123**, 207205 (2019).

34. Bose, A. *et al.* Tilted spin current generated by the collinear antiferromagnet ruthenium dioxide. *Nat. Electron.* **5**, 267–274 (2022).

35. Takeuchi, Y. *et al.* Spin-orbit torques in high-resistivity-W/CoFeB/MgO. *Appl. Phys. Lett.* **112**, 192408 (2018).

36. Sun, H. *et al.* Nonvolatile magnetization switching in a single-layer magnetic topological insulator. *Commun. Phys.* **6**, 222 (2023).

37. Chen, L. *et al.* Connections between spin-orbit torques and unidirectional magnetoresistance in ferromagnetic-metal–heavy-metal heterostructures. *Phys. Rev. B* **105**, L020406 (2022).

38. Han, B. *et al.* Determination of the Spin-Orbit Torques in Ferromagnetic–Heavy-Metal Bilayers Using Harmonic Longitudinal Voltage Measurements. *Phys. Rev. Appl.* **13**, 014065 (2020).

39. Avci, C. O. *et al.* Interplay of spin-orbit torque and thermoelectric effects in ferromagnet/normal-metal bilayers. *Phys. Rev. B* **90**, 224427 (2014).

40. Roschewsky, N. *et al.* Spin-orbit torque and Nernst effect in Bi-Sb/Co heterostructures. *Phys. Rev. B* **99**, 195103 (2019).

41. Togo, A., Shinohara, K. & Tanaka, I. Spglib: a software library for crystal symmetry search. *Sci. Technol. Adv. Mater. Methods* **4**, 2384822 (2024).

42. Belopolski, I. *et al.* Signatures of a time-reversal symmetric Weyl semimetal with only four Weyl points. *Nat. Commun.* **8**, 942 (2017).

43. Liu, T. *et al.* Crystallographically dependent bilinear magnetoelectric resistance in a thin WTe 2 layer. *Phys. Rev. B* **108**, 165407 (2023).

44. Marx, A. C. *et al.* Nonlinear magnetotransport in MoTe 2. *Phys. Rev. B* **109**, 125408 (2024).

45. Jafari, H., Roy, A. & Sławińska, J. Ferroelectric control of charge-to-spin conversion in WTe 2. *Phys. Rev. Mater.* **6**, L091404 (2022).

46. Hu, S. *et al.* Efficient perpendicular magnetization switching by a magnetic spin Hall effect in a noncollinear antiferromagnet. *Nat. Commun.* **13**, 4447 (2022).

47. Choi, G. S. *et al.* Highly Efficient Room-Temperature Spin-Orbit-Torque Switching in a Van der Waals Heterostructure of Topological Insulator and Ferromagnet. *Adv. Sci.* **11**, 2400893 (2024).

48. Wang, H. *et al.* Room temperature energy-efficient spin-orbit torque switching in two-dimensional van der Waals Fe3GeTe2 induced by topological insulators. *Nat. Commun.* **14**, 5173 (2023).

49. Stiehl, G. M. *et al.* Layer-dependent spin-orbit torques generated by the centrosymmetric transition metal dichalcogenide \ensuremath\beta\ensuremath-\mathrmMoTe_2. *Phys Rev B* **100**, 184402 (2019).

50. Xu, H. *et al.* High Spin Hall Conductivity in Large-Area Type-II Dirac Semimetal $PtTe_2$. *Adv. Mater.* **32**, 2000513 (2020).

51. Wang, X. *et al.* Room temperature field-free switching of CoFeB/MgO heterostructure based on large-scale few-layer WTe2. *Cell Rep. Phys. Sci.* **4**, 101468 (2023).

52. Wang, F. *et al.* Field-free switching of perpendicular magnetization by two-dimensional PtTe2/WTe2 van der Waals heterostructures with high spin Hall conductivity. *Nat. Mater.* **23**, 768–774 (2024).

53. Shin, I. *et al.* Spin–Orbit Torque Switching in an All-Van der Waals Heterostructure. *Adv. Mater.* **34**, 2101730 (2022).
